# The global, regional, and national prostate cancer burden and trends from 1990 to 2021, results from the global burden of disease study 2021

**DOI:** 10.3389/fpubh.2025.1553747

**Published:** 2025-05-21

**Authors:** Xiaomu Liu, Haiou Jiang

**Affiliations:** ^1^Department of Geriatrics, The First Hospital of China Medical University, Shenyang, China; ^2^Department of Neurology, The First Hospital of China Medical University, Shenyang, China; ^3^Shenyang Clinical Medical Research Center for Difficult and Serious Diseases of the Nervous System, Shenyang, China; ^4^Key Laboratory of Neurological Disease Big Data of Liaoning Province, Shenyang, China

**Keywords:** global burden of disease study, prostate cancer, prevalence, incidence, disability-adjusted life years, mortality

## Abstract

**Background:**

The rising annual incidence of prostate cancer (PCa) has become a significant health challenge for men worldwide. The study aims to estimate the contemporary disease burden of PCa across global, regional, and national levels.

**Methods:**

Data from the Global Burden of Disease Study 2021 (GBD 2021) was analyzed to evaluate trends in PCa incidence, prevalence, disability-adjusted life years (DALYs), and mortality from 1990 to 2021. Determinants of PCa burden were investigated through Spearman correlation analysis with socio-demographic index (SDI), decomposition analysis, and frontier analysis to assess regional improvement potential.

**Results:**

In 2021, global PCa incidence reached 1.32 million cases [95% uncertainty interval (UI): 1217320.93, 1400222.17]. Between 1990 and 2021, the global estimated annual percentage change (EAPC) of age-standardized incidence rates (ASIR), prevalence rates (ASPR), DALYs rates (ASDR), and mortality rates (ASMR) were declined or increased at −0.06% [95% confidence interval (CI): −0.21, 0.08], 0.42% (95% CI: 0.26, 0.58), −0.96% (95% CI: −1.05, −0.87), and −0.58% (95% CI: −0.73, −0.44), respectively. Low-middle SDI countries exhibited the steepest rate increases, with males over 50 years being most affected. Significant positive correlations emerged between SDI levels and ASIR (*R* = 0.543, *p* < 0.001) or ASPR (*R* = 0.709, *p* < 0.001), whereas EAPC of ASDR (*R* = −0.430, *p* < 0.001) or ASMR (*R* = −0.450, *p* < 0.001) inversely correlated with SDI among 204 countries and territories. Decomposition analysis revealed the global increase of DALYs for PCa was predominantly attributed to aging (77.65%) and population growth (58.59%). Frontier analysis identified substantial improvement potential across development spectra, particularly in middle to high SDI regions.

**Conclusion:**

Our findings demonstrated that despite modest declines in ASDR and ASMR within high SDI regions, PCa burden metrics persistently increased in low-middle SDI quintiles. Nations across all development levels require urgent implementation of evidence-based policies and precision management strategies to mitigate this growing public health challenge.

## 1 Introduction

Prostate cancer (PCa) ranks as the second most diagnosed malignancy and a leading cause of cancer-related mortality among men worldwide (1), with 1.4 million new cases and 375,304 deaths documented globally in 2020 (2). The past three decades have witnessed substantial transformations in PCa epidemiology, driven by innovations in diagnostic modalities, prostate-specific antigen (PSA) screening protocols, and persistent disparities in healthcare accessibility (3, 4). While high-income countries (HICs) have achieved mortality reduction through early detection and multimodal therapies, low- and middle-income countries (LMICs) face persistently elevated case fatality rates, highlighting systemic disparities in healthcare infrastructure, screening availability, and socioeconomic development (5, 6). This epidemiological divergence underscores the urgent need to re-evaluate the global burden of PCa through an equity-oriented framework, integrating demographic transitions, biological risk factors, and structural determinants into a cohesive analytical model.

The etiology of PCa is multifaceted, encompassing hereditary predisposition, age-related biological processes, hormonal pathways (e.g., androgen-mediated signaling), and modifiable lifestyle determinants, including obesity, physical inactivity, and dietary habits (7). Emerging evidence reveals a complex interaction between genetic ancestry and structural inequities: although African ancestry correlates with heightened PCa risk and tumor aggressiveness, mortality disparities among Black populations in HICs versus LMICs cannot be reductively attributed to genetic factors alone (8). Systemic inequalities in access to precision diagnostics, advanced radiotherapy, and novel therapies—such as Poly ADP-ribose Polymerase (PARP) inhibitors for metastatic castration-resistant PCa—profoundly exacerbate disparities in outcomes (9). Furthermore, sociocultural barriers such as limited health literacy and stigmatization of male health issues further contribute to delayed care-seeking in resource-constrained settings (10). Cumulatively, these insights highlight the complexity of PCa as a “disease of disparities,” demanding analytical frameworks that integrate structural socioeconomics with political determinants (11).

Contemporary research utilizing data from the Global Burden of Disease (GBD) study has identified key epidemiological trends in PCa. GBD 2019 analyses demonstrate that HICs exhibit higher incidence rates primarily attributable to PSA screening implementation, while LMICs demonstrate disproportionate mortality associated with advanced-stage detection and limited access to therapeutic innovations (3, 12). For instance, sub-Saharan African regions have experienced a marked mortality surge over the past three decades, despite documenting less than 10% of overall diagnosed cases (3). These disparities are further exacerbated by socioeconomic stratification, as quantified by the Socio-Demographic Index (SDI), a composite measure of income, educational attainment, and fertility rates (13, 14). HICs demonstrate heightened PCa incidence alongside reduced mortality, reflecting both the widespread adoption of PSA screening for early detection and subsequent improvements in survival outcomes. Conversely, LMICs—often constrained by fragmented healthcare infrastructure, limited diagnostic capacity, and low public health literacy—report heightened mortality rates, despite starkly lower incidence (15, 16). This paradoxical burden epitomizes the dual challenges of overdiagnosis in resource-abundant settings and systemic underdetection and undertreatment in underserved regions.

Despite significant advancements in PCa research, critical knowledge gaps persist in understanding its global burden. Existing epidemiological analyses predominantly emphasize incidence and mortality rates, often overlooking morbidity metrics such as disability-adjusted life years (DALYs), a key indicator of health system strain and disease impact (17). Furthermore, studies reliant on prior versions of the GBD datasets (e.g., 2019) have not incorporated recent shifts in screening guidelines, healthcare disruptions induced by the COVID-19 pandemic, or evolving demographic and socioeconomic trends. Additionally, while the SDI is acknowledged as a determinant of PCa burden, its nuanced association with epidemiological outcomes across diverse global regions remains insufficiently investigated. This study addresses these limitations by utilizing the comprehensive GBD 2021 database to evaluate global, regional, and national trends in PCa incidence, mortality, prevalence, and DALYs from 1990 to 2021. We further examine how sociodemographic development, as quantified by the SDI, modulates these outcomes across heterogeneous populations. By synthesizing the latest data and employing multidimensional epidemiological metrics, this work advances the epidemiological understanding of PCa’s temporal trajectory. The findings provide evidence-based strategic insights to inform policy formulation, emphasizing equitable resource allocation, optimized early-detection protocols, and targeted interventions to reduce premature mortality in high-burden populations.

## 2 Materials and methods

### 2.1 Data sources

Anonymized epidemiological data on PCa burden were obtained from the GBD 2021 database,^1^ hosted by the Institute for Health Metrics and Evaluation (IHME) at the University of Washington, United States (18). The GBD 2021 study employs a standardized methodological framework to quantify global, regional, and national trends in disease burden, injuries, and risk factors spanning from 1990 to 2021 (17). The methodological framework for GBD estimates has been extensively documented in peer-reviewed protocols (17, 19). Geographically, the GBD 2021 dataset encompasses 204 countries and territories, stratified into 21 distinct regions based on geographic proximity and epidemiological coherence. These regions are further classified into five SDI quintiles: Low, Low-middle, Middle, High-middle, and High SDI. The SDI quantifies sociodemographic development by combining three indicators: per capita income, average educational attainment among individuals aged 15 and older, and total fertility rate (20). This composite index ranges from 0 to 1, with higher values denoting greater sociodemographic advancement (21). Data inputs for PCa estimates in GBD 2021 were derived from a multidisciplinary array of sources, including nationally representative household surveys, civil registration and vital statistics systems, population-based censuses, cancer registry records, peer-reviewed literature, and disease surveillance reports. These sources underwent extensive quality assessment, hierarchical redistribution, and statistical correction to address biases and incompleteness, ensuring cross-country comparability.

### 2.2 Definition

In the GBD study, PCa is defined based on the International Classification of Diseases version 10 (ICD-10) diagnostic criteria: C61-C61.9, Z12.5, Z80.42, Z85.46 for mapping new cases, and C61-C61.9, D07.5, D29.1, D40.0 for mapping mortality (22).

### 2.3 Measurements

This study examined the burden of PCa across the global, regional (five SDI quintiles and 21 GBD regions), and national (204 countries and territories) levels from 1990 to 2021. Outcome measures included: (1) age-specific estimates for males aged 0 to 95+ years; (2) crude and age-standardized rates (ASRs) for incidence, prevalence, disability-adjusted life years (DALYs), and mortality. The incidence of PCa is estimated using a meta-analytical Bayesian regression tool named DisMod-MR version 2.1 (23); the prevalence of PCa is determined by summing new cases and previously diagnosed cases; DALYs for PCa are calculated by combining years of life lost and years lived with disability; the cause-specific mortality rates are modeled using the Cause of mortality Ensemble model (CODEm). All estimations are presented with 95% uncertainty interval (UI) to account for stochastic and systematic variability. Additionally, the mortality-to-incidence ratio (MIR) is calculated as a proxy for healthcare system efficacy by dividing the all-age crude mortality rate by the all-age crude incidence rate in males (24).

### 2.4 Change trends of PCa burden

The ASRs are a ratio that represents the expected number of cases per 100,000 people based on a standard population structure (19, 25). To quantify temporal trends in the PCa burden from 1990 to 2021, the estimated annual percentage change (EAPC) was computed for the following age-standardized incidence rate (ASIR), age-standardized prevalence rate (ASPR), age-standardized DALY rate (ASDR), and age-standardized rates of mortality (ASMR). Specifically, the EAPC is calculated via a log-linear regression model: Ln (ASR) = *α* + *βx*+ɛ, where Ln(ASR) represents the natural logarithm of the ASR, α denotes the expected log of ASR when *x* = 0, β represents the average annual change in the log of ASR, *x* signifies the number of years elapsed from the baseline year, and ɛ represents the random error term. The EAPC is calculated as: EAPC = 100 × (exp (β) − 1), with the corresponding 95% confidence interval (CI) estimated from the standard error of the EAPC estimate. A 95% CI > 0 indicates a statistically significant upward trend, a 95% CI < 0 indicates a statistically significant downward trend, a 95% CI including 0 suggests no statistically significant trend (26).

### 2.5 Decomposition analysis

Decomposition analysis was conducted to quantify the contributions of three demographic drivers—population aging, population growth, and epidemiological changes—to temporal shifts in the DALYs of PCa. The analysis employed the following formula (27):


$${\mathrm{DALYs}}_{ax,px,ex}=\sum_{i=1}^{20}(a_{i,x}*p_{x}*e_{i,x})$$

Where DALYs*_ax,px,ex_* represents DALYs attributable to aging, population growth, and epidemiologic changes in year *x*, the a*_i,x_* represents the proportion of the population in age group *i* at year *x*, the *p_x_* denotes the population size at year *x*, and *e_i,x_* represents the DALYs rate for a specific age group *i* at year *x*. By evaluating the impact of altering one component while holding the others constant, the influence of each component on changes of DALYs is determined.

### 2.6 Frontier analysis

Frontier analysis was utilized to evaluate the relationship between sociodemographic development and ASDR for PCa (28). A data envelopment analysis was conducted using the free disposal hull method to create non-linear frontiers for ASDR by SDI, based on PCa data from 1990 to 2021. To address uncertainty, we generated 1,000 bootstrapped samples through random sampling with replacement from all countries and territories across all study years. The mean DALYs for PCa at each SDI value from these samples were computed. To analyze how the ASDR compared to the frontier in 2021, we calculated the effective difference, defined as the absolute distance from the frontier, using 2021 SDI and PCa DALYs data for each country or territory. The distance from this DALYs frontier is referred to as the effective difference: a larger effective difference indicates greater gaps between observed outcomes and achievable benchmarks given a country’s SDI level (29).

### 2.7 Statistical analysis

All statistical analyses were performed using the R version 4.4.2 (R foundation for Statistical Computing, Vienna, Austria). The “ggplot2” package was applied to depict graphs and charts, while the “maps” package was used to render the globe. A *p*-value < 0.05 (two-tailed) was considered statistically significant.

### 2.8 Ethics statement

The Institutional Review Board of the First Hospital of China Medical University determined that the ethical approval was not required for this study, as it exclusively analyzed publicly available, aggregated, and anonymized data. This study adhered to the Strengthening the Reporting of Observational Studies in Epidemiology (STROBE) reporting guidelines.

## 3 Results

### 3.1 Global burden of PCa in 1990 and 2021

**Figure 1:**
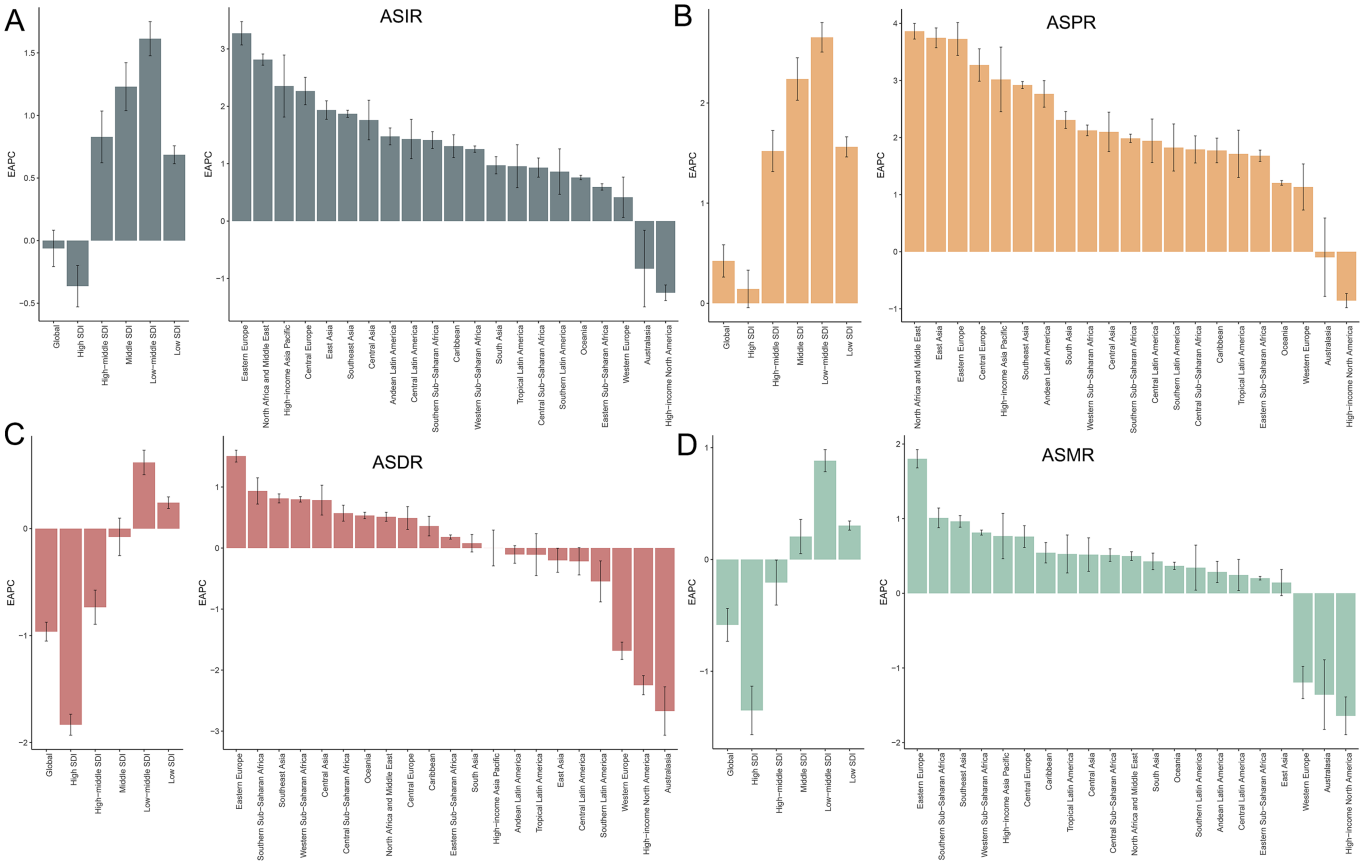
Temporal trends in EAPC of ASIR **(A)**, ASPR **(B)**, ASDR **(C)**, and ASMR **(D)** for PCa across global, SDI quintiles, and 21 GBD regions from 1990 to 2021. SDI, socio-demographic index; ASIR, age-standardized incidence, ASPR, age-standardized prevalence, ASDR, age-standardized disability-adjusted life years; ASMR, age-standardized mortality; PCa, prostate cancer; EAPC, estimated annual percentage change; PCa, prostate cancer; GBD, Global Burden of Disease; SDI, socio-demographic index.

In 1990, the global incidence of PCa was approximately 0.51 million (95% UI: 480851.38, 524697.41) cases, with an ASIR of 32.64 (95% UI: 30.86, 33.86) per 100,000 population. By 2021, this had increased to approximately 1.32 million (95% UI: 1217320.93, 1400222.17) cases, with an ASIR of 34.05 (95% UI: 31.27, 36.00) per 100,000 population (Table 1). Between 1990 and 2021, the global number of incidence cases of PCa increased by 161.53% (95% UI: 150.37, 172.47), while the corresponding ASIR remained stable with an EAPC of −0.06 (95% CI: −0.21, 0.08) (Table 1; Figure 1A). Supplementary Table S1 showed that the prevalence of PCa cases increased from 3.6 million (95% UI: 3445436.84, 3705436.00) in 1990 to 10.39 million (9705680.48, 10904399.86) in 2021, representing an increase of 188.85% (95% UI: 176.52, 200.98). Furthermore, the ASPR increased from 218.33 (95% UI: 208.48, 225.67) in 1990 to 260.05 (95% UI: 243.39, 272.68) in 2021, with an EAPC of 0.42% (95% CI: 0.26, 0.58) during this period (Figure 1B). Supplementary Table S2 revealed that the global DALYs of PCa were 8.14 million (95% UI: 7177066.89, 8809184.40) in 2021, representing a 96.34% (95% UI: 86.16, 107.71) increase compared to 4.15 million cases in 1990 (95% UI: 3754315.11, 4401619.73). Conversely, the trend in ASDR gradually decreased over the past 30 years, with an EAPC of −0.96 (95% CI: −1.05, −0.87) (Figure 1C). Additionally, as shown in Supplementary Table S3, PCa-related mortality was 0.21 million (95% UI: 194221.85, 224327.73) in 1990 and 0.43 million (95% UI: 381872.79, 463645.28) in 2021, increased by 104.02% (95% UI: 92.73, 116.11). The corresponding ASMR decreased from 16.35 (95% UI: 15.02, 17.28) in 1990 to 12.63 (95% UI: 11.16, 13.55) in 2021, with an EAPC of −0.58 (95% CI: −0.73, −0.44) over the three decades (Figure 1D). Correspondingly, the global MIR reduced from 0.42 in 1990 to 0.33 in 2021 (Supplementary Table S4).

**Table 1:** The global incidence of PCa and its estimated trends from 1990 to 2021.

Characteristics	1990		2021		1990–2021	1990–2021
	Incidence cases No. (95% UI)	ASIR per 100,000 No. (95% UI)	Incidence cases No. (95% UI)	ASIR per 100,000 No. (95% UI)	Cases change (95% UI)	EAPC (95% CI)
Global	506405.20 (480851.38, 524697.41)	32.64 (30.86, 33.86)	1324382.90 (1217320.93, 1400222.17)	34.05 (31.27, 36.00)	161.53 (150.37, 172.47)	−0.06 (−0.21, 0.08)
High SDI	339223.86 (324610.12, 348526.37)	73.74 (70.21, 75.85)	694560.94 (647353.23, 727638.02)	70.92 (66.29, 74.22)	104.75 (95.48, 112.46)	−0.36 (−0.53, −0.20)
High-middle SDI	83918.43 (79122.21, 87906.65)	22.76 (21.31, 23.86)	268333.28 (238028.00, 291705.97)	30.36 (26.96, 33.04)	219.75 (192.02, 245.02)	0.83 (0.62, 1.04)
Middle SDI	46618.40 (39994.07, 51526.26)	12.49 (10.80, 13.93)	228112.47 (194579.64, 258723.26)	19.43 (16.51, 22.07)	389.32 (344.60, 445.53)	1.23 (1.04, 1.42)
Low-middle SDI	23184.73 (18103.16, 26834.72)	9.46 (7.46, 11.07)	95746.38 (79598.90, 111125.38)	15.90 (13.29, 18.51)	312.97 (255.95, 382.70)	1.61 (1.48, 1.75)
Low SDI	12827.72 (8388.83, 16190.19)	14.50 (9.59, 18.27)	35707.43 (23033.94, 44175.39)	18.14 (11.84, 22.34)	178.36 (142.23, 231.97)	0.69 (0.61, 0.76)
Andean Latin America	2233.64 (1800.70, 2784.44)	26.12 (21.28, 32.23)	11257.16 (8196.43, 15488.00)	42.16 (30.78, 57.66)	403.98 (293.02, 546.20)	1.47 (1.33, 1.62)
Australasia	11154.93 (10153.20, 11894.68)	110.31 (100.67, 117.87)	25372.68 (21471.29, 29883.63)	97.88 (82.70, 115.15)	127.46 (94.06, 171.55)	−0.83 (−1.49, −0.16)
Caribbean	7122.13 (6577.25, 7884.29)	60.25 (55.66, 66.69)	23250.52 (19899.05, 26780.01)	93.77 (80.45, 108.03)	226.45 (177.62, 279.82)	1.30 (1.11, 1.50)
Central Asia	1858.99 (1732.72, 1976.49)	11.25 (10.46, 11.95)	4640.40 (4156.91, 5157.17)	14.90 (13.51, 16.48)	149.62 (120.94, 179.87)	1.76 (1.42, 2.11)
Central Europe	14514.28 (13746.88, 15353.56)	24.68 (23.35, 26.18)	47883.31 (43406.04, 52543.98)	48.90 (44.40, 53.65)	229.90 (199.74, 262.68)	2.27 (2.03, 2.50)
Central Latin America	12882.18 (12255.84, 13390.85)	36.04 (34.18, 37.51)	72324.58 (62261.12, 83103.36)	65.04 (56.10, 74.53)	461.43 (378.04, 543.26)	1.43 (1.09, 1.77)
Central Sub-Saharan Africa	1504.26 (965.18, 1986.52)	21.18 (13.81, 28.59)	4527.77 (2736.57, 6190.97)	28.26 (17.17, 39.13)	201.00 (128.71, 303.03)	0.93 (0.77, 1.10)
East Asia	14813.84 (11139.17, 18812.63)	5.13 (3.93, 6.62)	97023.93 (71371.94, 129638.08)	9.90 (7.39, 13.06)	554.95 (386.80, 756.59)	1.93 (1.77, 2.10)
Eastern Europe	20794.95 (19663.20, 21763.71)	22.80 (21.55, 23.82)	76061.26 (68013.84, 83465.93)	56.28 (50.56, 61.65)	265.77 (225.45, 303.68)	3.27 (3.07, 3.48)
Eastern Sub-Saharan Africa	5387.43 (3231.93, 6870.49)	18.28 (11.12, 23.18)	14544.49 (9312.75, 18606.79)	22.55 (14.67, 28.24)	169.97 (122.76, 246.83)	0.59 (0.54, 0.65)
High-income Asia Pacific	10826.95 (10154.92, 11450.19)	14.62 (13.61, 15.46)	65589.02 (57662.78, 72526.35)	28.74 (25.29, 31.70)	505.79 (437.26, 567.63)	2.35 (1.81, 2.89)
High-income North America	198195.98 (189548.91, 204980.91)	130.27 (124.25, 134.72)	316171.44 (297651.78, 329966.49)	101.92 (95.89, 106.40)	59.52 (51.87, 67.27)	−1.25 (−1.38, −1.11)
North Africa and Middle East	8445.10 (6245.10, 10445.33)	12.17 (9.10, 15.41)	55933.69 (39216.99, 67925.42)	27.39 (19.27, 33.39)	562.32 (450.39, 703.47)	2.81 (2.72, 2.91)
Oceania	198.10 (136.74, 271.11)	19.98 (13.90, 27.26)	669.52 (426.80, 930.51)	24.56 (15.95, 34.27)	237.97 (164.21, 357.62)	0.76 (0.72, 0.80)
South Asia	11974.02 (8454.41, 14641.37)	5.12 (3.63, 6.31)	48149.74 (39399.66, 65765.30)	7.60 (6.26, 10.42)	302.12 (209.78, 432.40)	0.97 (0.82, 1.12)
Southeast Asia	8732.67 (6066.72, 10198.68)	9.41 (6.59, 11.07)	43150.44 (28220.96, 53091.34)	16.72 (10.97, 20.71)	394.13 (297.66, 499.62)	1.87 (1.81, 1.93)
Southern Latin America	6.30 (5.63, 7.02)	33.35 (29.78, 37.11)	16.82 (14.36, 19.47)	44.06 (37.83, 50.95)	167.14 (122.40, 212.48)	0.86 (0.47, 1.26)
Southern Sub-Saharan Africa	3.43 (2.47, 4.57)	36.91 (26.81, 48.80)	10.87 (8.12, 12.87)	55.12 (40.52, 64.65)	217.17 (148.26, 321.58)	1.41 (1.26, 1.56)
Tropical Latin America	10.38 (9.81, 10.87)	28.68 (26.85, 30.11)	45.43 (42.31, 48.15)	41.32 (38.45, 43.85)	337.71 (309.84, 368.00)	0.95 (0.58, 1.33)
Western Europe	145.85 (138.96, 151.81)	61.06 (58.11, 63.50)	314.97 (285.54, 338.63)	73.05 (66.40, 78.43)	115.96 (98.47, 132.50)	0.41 (0.06, 0.77)
Western Sub-Saharan Africa	9.81 (5.64, 12.93)	29.10 (17.10, 37.82)	29.74 (15.63, 40.05)	41.46 (21.70, 55.16)	202.99 (130.29, 302.53)	1.25 (1.20, 1.31)

ASIR, age-standardized incidence; CI, confidence interval; PC, percentage change; EAPC, estimated annual percentage change; UI, uncertainty interval; PCa, prostatic cancer.

### 3.2 PCa burden by SDI quintiles

**Figure 2:**
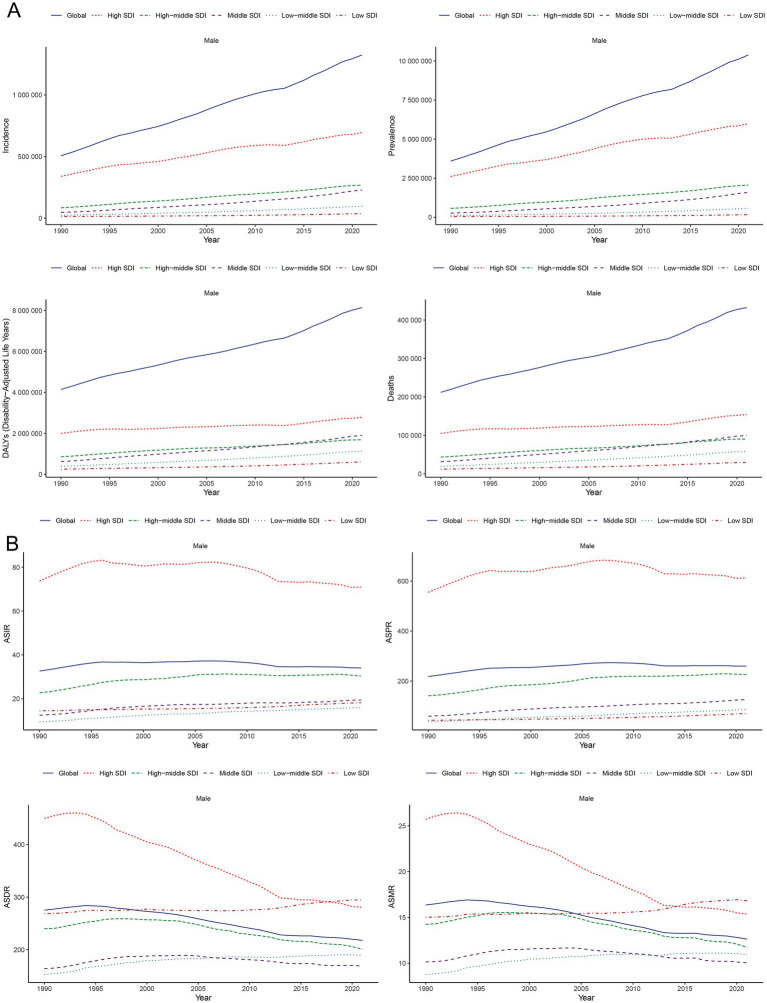
Temporal trends of incidence, prevalence, DALYs, and mortality **(A)** and their corresponding ASIR, ASPR, ASDR, and ASMR **(B)** for PCa in global and SDI quintiles from 1990 to 2021. SDI, socio-demographic index; ASIR, age-standardized incidence; ASPR, age-standardized prevalence; ASDR, age-standardized disability-adjusted life years; ASMR, age-standardized mortality; DALYs, disability-adjusted life years; PCa, prostate cancer; SDI, socio-demographic index.

Figure 2A illustrates the gradual increase in incidence, prevalence, DALYs, and mortality of PCa across all SDI quintiles from 1990 to 2021, with High SDI quintile exhibiting the highest cases of incidence of PCa (694560.94; 95% UI: 647353.23, 727638.02), prevalence (5987871.576; 95% UI: 5660940.43, 6245000.12), DALYs (2788077.76; 95% UI: 2562127.53, 2985290.67), and mortality (154422.87; 95% UI: 138844.11, 163654.78) in 2021 (Table 1). Regarding ASR, although Middle, Low-middle, and Low SDI quintile demonstrated increases in ASIR and ASPR over the past three decades, High SDI quintile still had the most ASIR (70.92; 95% UI: 66.29, 74.22) and ASPR (612.57; 95% UI: 579.53, 638.50) for PCa (Figure 2B; Table 1; Supplementary Table S1) in 2021. Increasing trends of ASDR and ASMR were observed in Low-middle, and Low SDI regions between 1990 and 2021 (Figure 2B), with the highest ASDR (294.26; 95% UI: 189.52, 363.32) and ASMR (16.80; 95% UI: 10.86, 20.59) for PCa observed in low SDI quintile in 2021. With respect to EAPC, the ASIR of PCa increased in High-middle, Middle, Low-middle, and Low SDI quintiles, with the most significant upward trends in Low-middle SDI regions (EAPC = 1.61; 95% CI: 1.48, 1.75), while decreasing in High SDI quintile (EAPC = −0.36; 95% CI: −0.53, −0.20) (Table 1; Figure 1A). The ASPR of PCa increased in all SDI quintiles, with the largest increase in low-middle SDI regions (EAPC = 2.66; 95% CI: 2.51, 2.80) (Supplementary Table S1; Figure 1B). Additionally, the ASDR of PCa increased in Low-middle and Low SDI quintiles, while decreasing in High and High-middle quintiles (Supplementary Table S2; Figure 1C). An upward trend in ASMR of PCa was found in Middle, Low-middle and Low SDI quintiles, a downward trend in High SDI quintile, and a stable trend in High-middle SDI (Supplementary Table S3; Figure 1D). The Low-middle SDI quintile exhibited the highest increase in ASDR (EAPC = 0.62; 95% CI: 0.50, 0.73) and ASMR (EAPC = 0.88; 95% CI: 0.78, 0.98), while High SDI quintile demonstrated the most pronounced declining trends in ASDR (EAPC = −1.83; 95% CI: −1.93, −1.73) and ASMR (EAPC = −1.35; 95% CI: −1.57, −1.13) (Figures 1C,D; Supplementary Tables S2, S3) between 1990 and 2021. Notably, MIR in 2021 ranged from 0.22 in the High SDI quintile to 0.82 in the Low SDI quintile, reflecting disparities in early detection and treatment efficacy (Supplementary Table S4).

### 3.3 GBD regional burden of PCa

**Figure 3:**
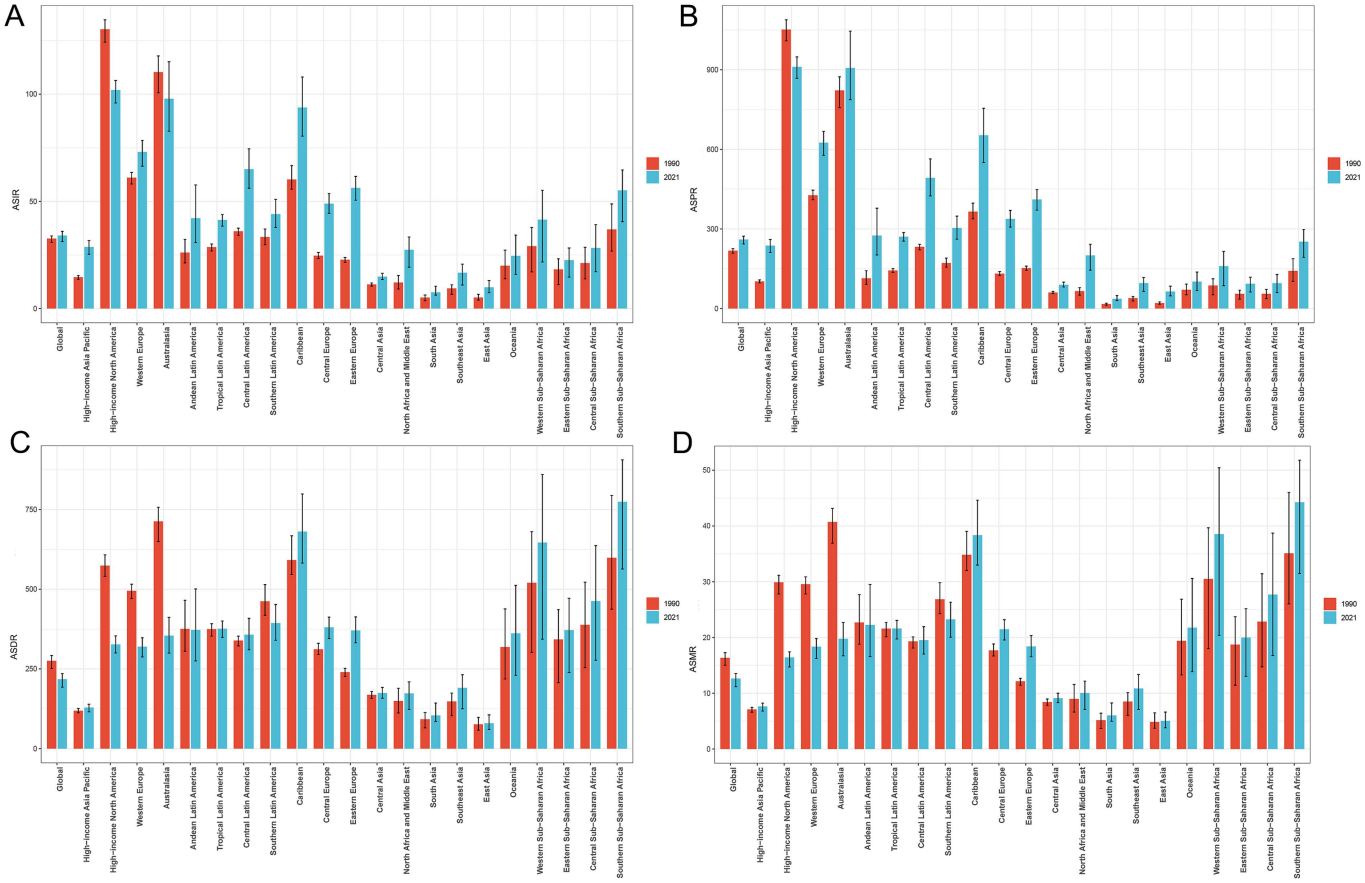
Comparison of ASIR **(A)**, ASPR **(B)**, ASDR **(C)**, and ASMR **(D)** of PCa in global and 21 GBD regions in 1990 versus 2021. ASIR, age-standardized incidence; ASPR, age-standardized prevalence; ASDR, age-standardized disability-adjusted life years; ASMR, age-standardized mortality; PCa, prostate cancer; GBD, Global Burden of Disease.

Compared to 1990, the incidence, prevalence, DALYs, and mortality cases of PCa increased across all 21 GBD regions in 2021 (Table 1; Supplementary Tables S1–S3). High-income North America exhibited the highest incidence of PCa in 2021 (316171.44; 95% UI: 297651.78, 329966.49) and the highest ASIR (101.92; 95% UI: 95.89, 106.40) (Table 1; Figure 3A). Most GBD regions showed an upward trend in ASIR, with Eastern Europe exhibiting the most rapid increase in ASIR (EAPC = 3.27; 95% CI: 3.07, 3.48) (Table 1; Figure 1A). In terms of ASPR for PCa, High-income North America ranked first (910.62; 95% UI: 867.85, 948.35) (Supplementary Table S1; Figure 3B), and North Africa and Middle East experienced the most significant increase in ASPR (EAPC = 3.86; 95% CI: 3.73, 4.00) (Supplementary Table S1; Figure 1B). Furthermore, nearly half of the GBD regions showed increased ASDR between 1990 and 2021, with Southern Sub-Saharan Africa exhibiting the highest ASDR (774.40; 95% UI: 563.55, 905.89) (Supplementary Table S2; Figure 3C) and Eastern Europe demonstrating the fastest growth of ASDR, with an EAPC of 1.51 (95% CI: 1.41, 1.60) (Supplementary Table S2; Figure 1C). Although ASMRs declined in most GBD regions, Southern Sub-Saharan Africa retained the highest ASMR (44.25; 95% UI: 31.48, 51.79) (Supplementary Table S3; Figure 3D) and Eastern Europe experienced the most rapid growth rate in ASMR (EAPC = 1.80, 95% CI: 1.68, 1.92) (Supplementary Table S3; Figure 1D). Conversely, the most pronounced ASRs declines occurred in Australasia and High-income North America (Figures 1A–D; Table 1; Supplementary Tables S1–S3). Notably, in 2021, MIR starkly differed by development status: MIR in high-income regions (High-income North America 0.16, Australasia 0.21, and Western Europe 0.27) were markedly lower than in low-income regions (Western Sub-Saharan Africa 0.83, Central Sub-Saharan Africa 0.88, and Eastern Sub-Saharan Africa 0.79) in 2021 (Supplementary Table S4).

### 3.4 PCa burden by global countries and territories

**Figure 4:**
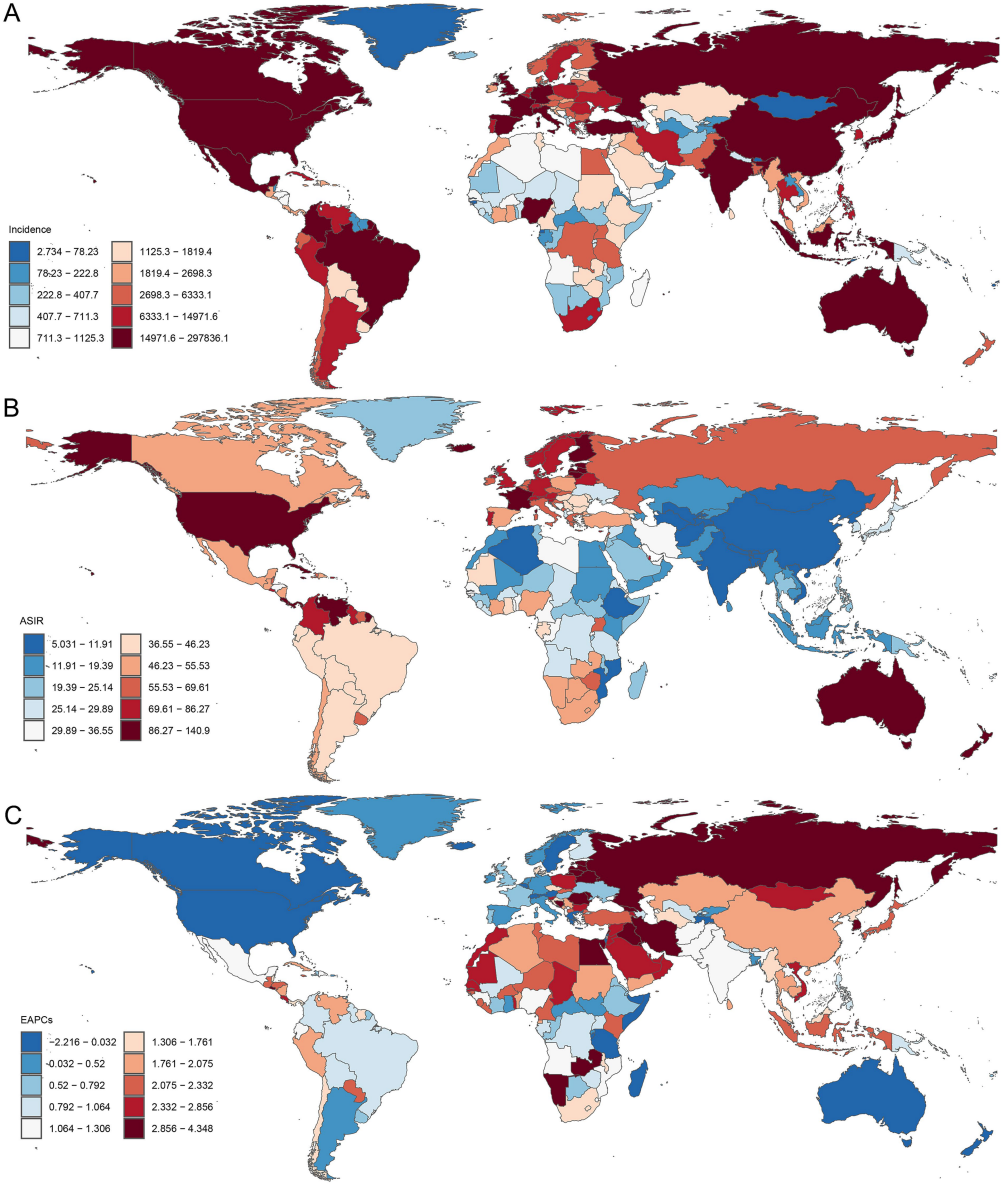
Global distribution of PCa incidence burden across 204 countries and territories. **(A)** The incidence of PCa in 2021; **(B)** ASIR in 2021; **(C)** EAPC in ASIR from 1990 to 2021. ASIR, age-standardized incidence; EAPC, estimated annual percentage change; PCa, prostate cancer.

Geospatial analyses of 204 countries and territories in 2021 revealed substantial disparities in the PCa burden (Figure 4). The America reported the highest number of incidence cases (297836.07; 95% UI: 280073.42, 311399.89) (Figure 4A), prevalence (2671778.55; 95% UI: 2547382.78, 32786354.04) (Supplementary Figure S1A), DALY (912428.2919; 95% UI: 833910.6395; 989998.6664) (Supplementary Figure S2A), and mortality (44032.37; 95% UI: 39406.012, 46728.34) (Supplementary Figure S3A). In 2021, the ASRs of PCa varied notably among different countries and territories, Bermuda recorded the highest ASIR (196.12; 95% UI: 154.67, 247.67) (Figure 4B) and ASPR (1527.67; 95% UI: 1206.84, 1915.59) of PCa (Supplementary Figure S2B), while Grenada had the highest ASDR (1542.79; 95% UI: 1349.059, 1750.86) (Supplementary Figure S2B) and ASMR (93.90; 95% UI: 83.06, 105.50) (Supplementary Figure S3B). From 1990 to 2021, ASIR and ASPR increased in cost countries and territories. The fastest ASIR rises were observed in South Korea (EAPC = 4.35, 95% CI: 3.74, 4.96), Georgia (EAPC = 4.30, 95% CI: 3.55, 5.06), and Republic of Estonia (EAPC = 4.14, 95% CI: 3.40, 4.88) (Figure 4C). Similarly, South Korea led ASPR growth (EAPC = 5.89, 95% CI: 5.16, 6.61), followed by Estonia (EAPC = 4.77, 95% CI: 4.06, 5.49), and Lebanon (EAPC = 4.63, 95% CI: 4.29, 4.97) (Supplementary Figure S1C). Furthermore, ASDR and ASMR increased in nearly half of the countries and territories, with the most rapid increase in ASDR reported in Georgia (EAPC = 3.89, 95% CI: 3.10, 4.68), Republic of Zambia (EAPC = 2.58, 95% CI: 2.23, 2.93), and Arab Republic of Egypt (EAPC = 2.47, 95% CI: 2.18, 2.77) (Supplementary Figure S2C). The largest increases in ASMR were observed in Georgia (EAPC = 4.07, 95% CI: 3.18, 4.97), Arab Republic of Egypt (EAPC = 2.58, 95% CI: 2.24, 2.93), and Republic of Latvia (EAPC = 2.36, 95% CI: 2.06, 2.65) (Supplementary Figure S3C). In contrast, ASDR and ASMR declined in the remaining countries (Supplementary Figures S2C, S3C), with the largest decreases in ASDR observed in Canada (EAPC = −3.27; 95% CI: −3.55, −3.00), Australia (EAPC = −2.80; 95% CI: −3.26, −2.34), and Swiss Confederation (EAPC = −2.53; 95% CI: −2.67, −2.39) (Supplementary Figure S2C). The largest decreasing trends in ASMR were observed in Canada (EAPC = −3.15; 95% CI: −3.37, −2.92), Australia (EAPC = −2.90; 95% CI: −3.28, −2.51), and Kingdom of Belgium (EAPC = −2.75; 95% CI: −2.91, −2.58) (Supplementary Figure S3C).

### 3.5 Burden of PCa by different age groups

**Figure 5:**
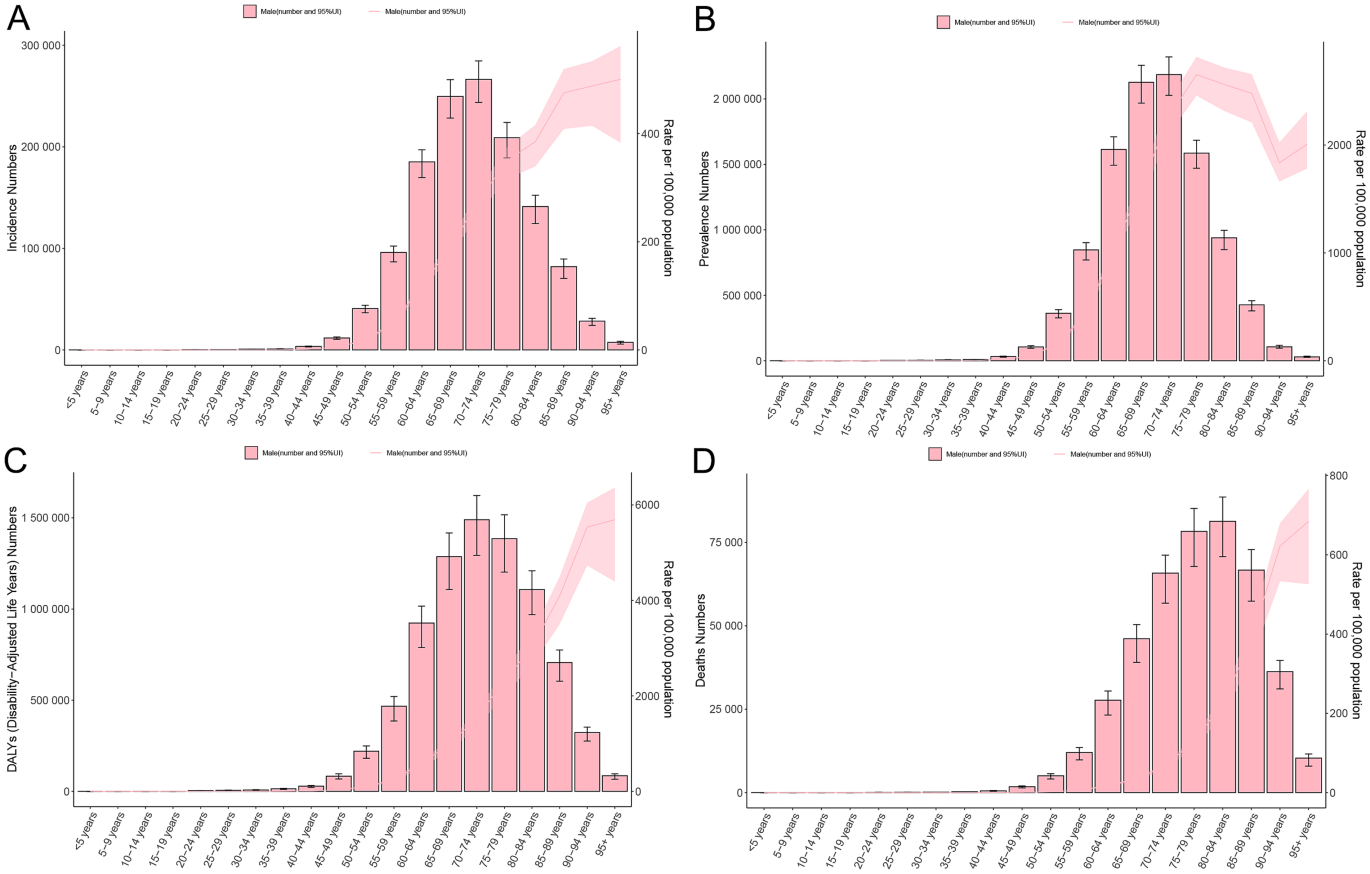
Age-specific burden of PCa in 2021. Crude rate and total number of incidence cases **(A)**, prevalence cases **(B)**, DALYs **(C)**, and mortality **(D)**. DALYs, disability-adjusted life years; PCa, prostate cancer; UI, uncertainty interval.

Age-specific patterns of PCa burden among males were illustrated in Figure 5. In 2021, the incidence and prevalence were predominantly found in males aged over 50 years, particularly in the 70–74 age group (266469.55; 95% UI: 243753.54, 284649.43; and 2185580.80; 95% UI: 2027158.70, 2320238.50, respectively). Furthermore, the trends of incidence and prevalence rates sharply increased with age beyond 50 years, peaking in the oldest cohort (>95 years) for incidence (500.25; 95% UI: 383.17, 561.71) (Figure 5A) and in the 75–79 age group for prevalence (2652.98; 95% UI: 2459.44, 2816.69) (Figure 5B). Similarly, the numbers of DALYs and mortality were concentrated in males aged above 50 years. The 70–74 age group bore the highest DALY burden (1489614.12; 95% UI: 1293844.93, 1622297.81), while the 80–84 age group recorded the highest mortality (81289.90; 95% UI: 70710.22, 88593.94) (Figures 5C,D). The corresponding DALYs and mortality rates gradually increased with advancing age, reaching maximal levels in males above 95 years (5690.86; 95% UI: 4394.10, 6365.24; and 684.24; 95% UI: 525.93, 766.45, respectively) (Figures 5C,D).

### 3.6 Correlation analysis of PCa burden and SDI

**Figure 6:**
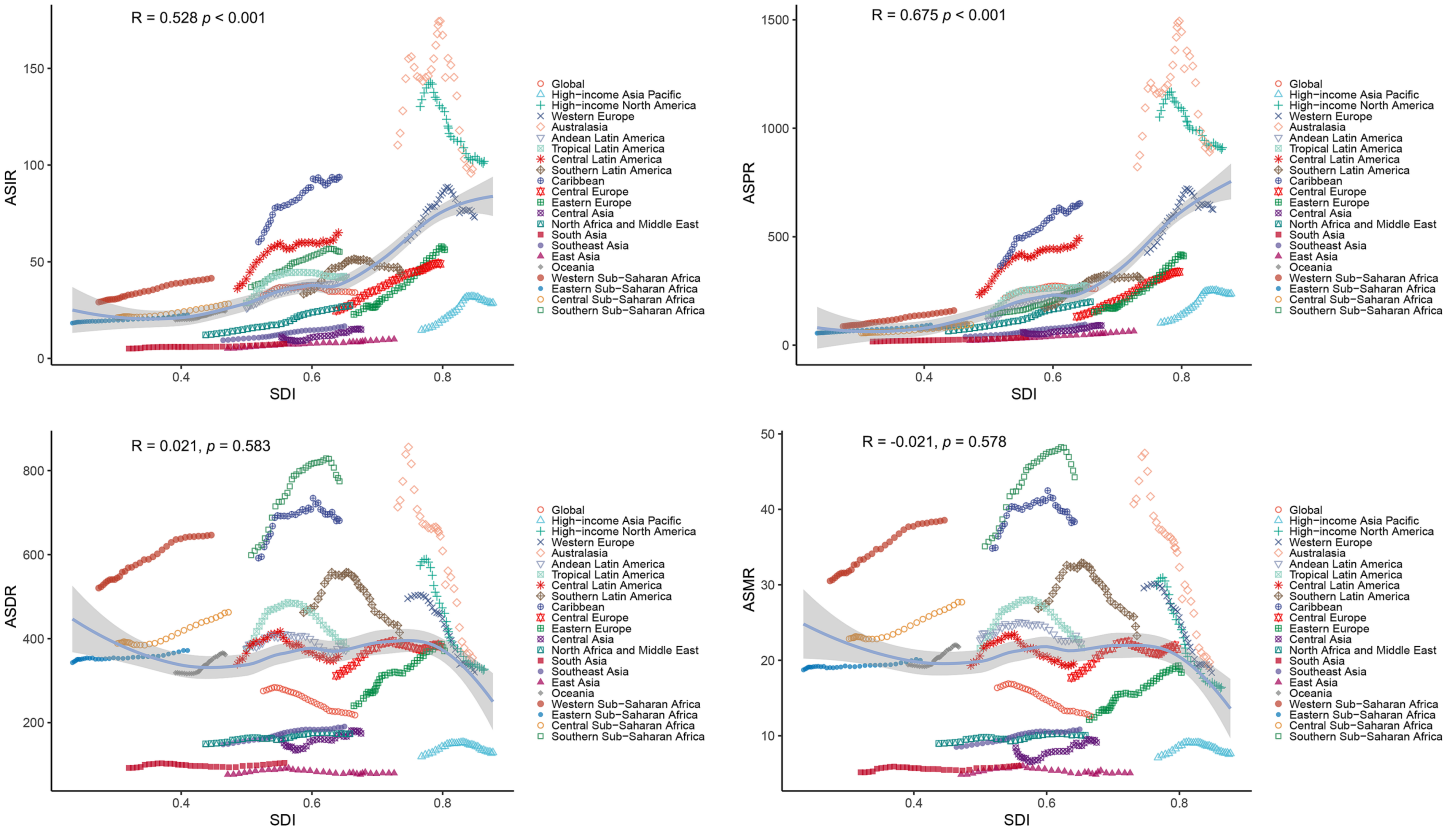
Correlations between SDI and age-standardized rates (ASIR, ASPR, ASDR, ASMR) of PCa across 21 GBD regions from 1990 to 2021. ASR, age-standardized rates; DALYs, disability-adjusted life years; PCa, prostate cancer; ASIR, age-standardized incidence; ASPR, age-standardized prevalence; ASDR, age-standardized disability-adjusted life years; ASMR, age-standardized mortality; GBD, Global Burden of Disease; SDI, socio-demographic index.

**Figure 7:**
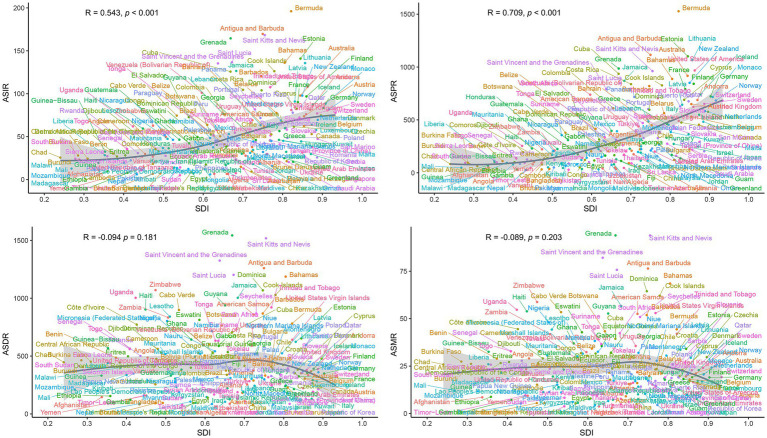
National-level associations between SDI and age-standardized rates (ASIR, ASPR, ASDR, ASMR) of PCa in 2021. ASR, age-standardized; DALYs, disability-adjusted life years; PCa, prostate cancer; ASIR, age-standardized incidence; ASPR, age-standardized prevalence; ASDR, age-standardized disability-adjusted life years; ASMR, age-standardized mortality; SDI, socio-demographic index.

**Figure 8:**
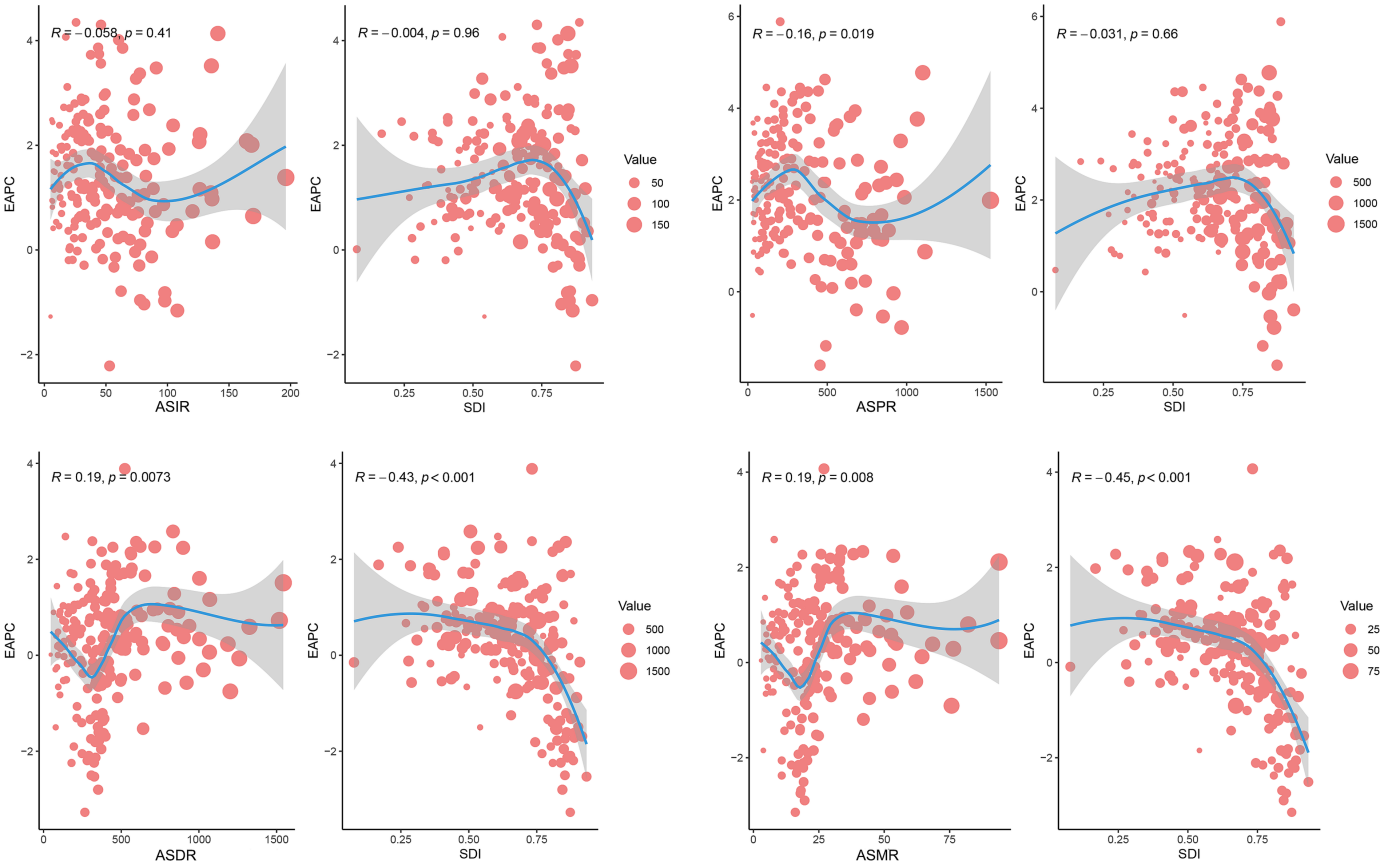
Correlation analysis between SDI and EAPCs of ASIR, ASPR, ASDR, or ASMR for PCa across 204 countries and territories in 2021. Dot size reflects case counts. DALYs, disability-adjusted life years; PCa, prostate cancer; ASIR, age-standardized incidence; ASPR, age-standardized prevalence; ASDR, age-standardized disability-adjusted life years; ASMR, age-standardized mortality.

Correlation analysis revealed distinct associations between PCa burden metrics and SDI. A significant positive linear correlation was observed between SDI and ASIR (*R* = 0.528, *p* < 0.001) or ASPR (*R* = 0.675, *p* < 0.001) (Figure 6). In contrast, ASDR and ASMR of PCa demonstrated no significant associations with SDI (Figure 6). Similarly, at the global level, ASIR and ASPR exhibited strong linear relationships with SDI levels among 204 countries and territories (*R* = 0.543, *p* < 0.001 and *R* = 0.709, *p* < 0.001, respectively) (Figure 7). However, ASDR and ASMR again showed no significant association with SDI (Figure 7). Analyses of EAPCs in relation to SDI yielded mixed findings: EAPCs for ASIR and ASPR across most countries were above zero but showed no significant correlation with SDI (*R* = −0.004, *p* = 0.96 and *R* = −0.031, *p* = 0.66, respectively) (Figure 8). Furthermore, a V-shaped relationship was identified between ASDR or ASMR and their corresponding EAPCs (*R* = 0.19, *p* = 0.0073 and *R* = 0.19, *p* = 0.008, respectively), and EAPCs for ASDR and ASMR were parabolically correlated with SDI (*R* = −0.43, *p* < 0.001 and *R* = −0.45, *p* < 0.001, respectively), indicating accelerated declined in high-SDI regions.

### 3.7 Decomposition analysis in DALYs

**Figure 9:**
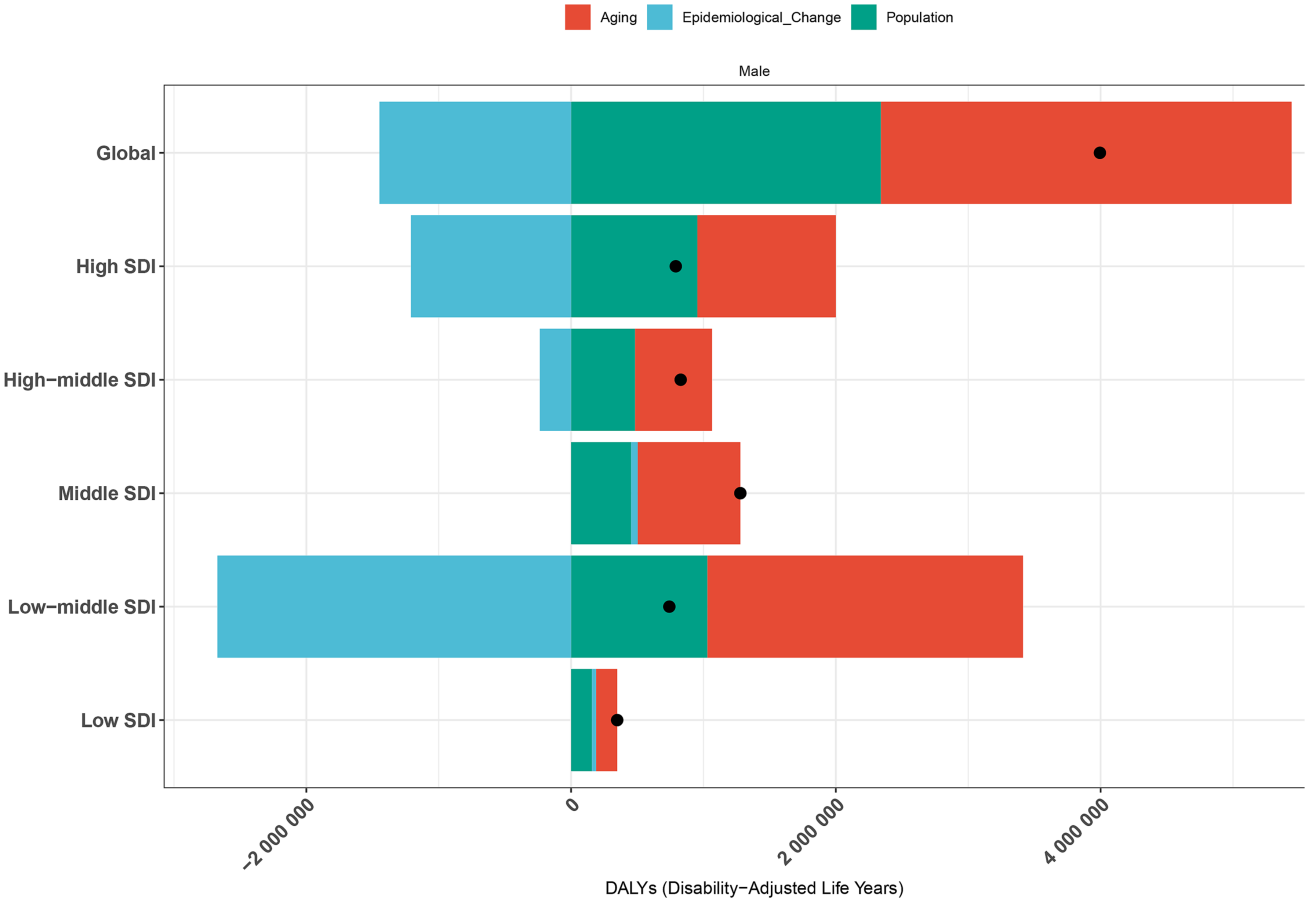
Decomposition analysis of PCa-related DALYs by population growth, aging, and epidemiological trends in worldwide and across SDI quintiles from 1990 to 2021. The black dot represents total net change; positive values indicate contributing increases to DALYs. DALYs, disability-adjusted life years; PCa, prostate cancer; SDI, socio-demographic index.

Decomposition analysis of DALYs attributed to PCa between 1990 and 2021 revealed distinct demographic and epidemiological contributions (Figure 9; Supplementary Table S5). Globally, population aging accounted for 77.65% of the increased burden of DALYs, followed by population growth (58.59%), while epidemiological changes contributed to −36.24%. Across SDI quintiles, aging and population growth exerted positively contributions to DALYs throughout the study period. The Low-middle SDI quintile recorded the largest contribution from aging (321.24%), followed by the High SDI quintile (132.34%). Similarly, population growth had the most substantial impact in the Low-middle SDI quintile (138.72%), followed by the High SDI quintile (120.59%). In contrast, epidemiological changes exhibited heterogeneous patterns. Middle and Low SDI quintiles experienced increases in DALYs due to epidemiological shifts. Whereas, High, High-middle, and Low-middle SDI quintiles demonstrated declines, with the most pronounced reduction observed in the Low-middle SDI quintile (−359.96%).

### 3.8 Frontier analysis of ASDR

**Figure 10:**
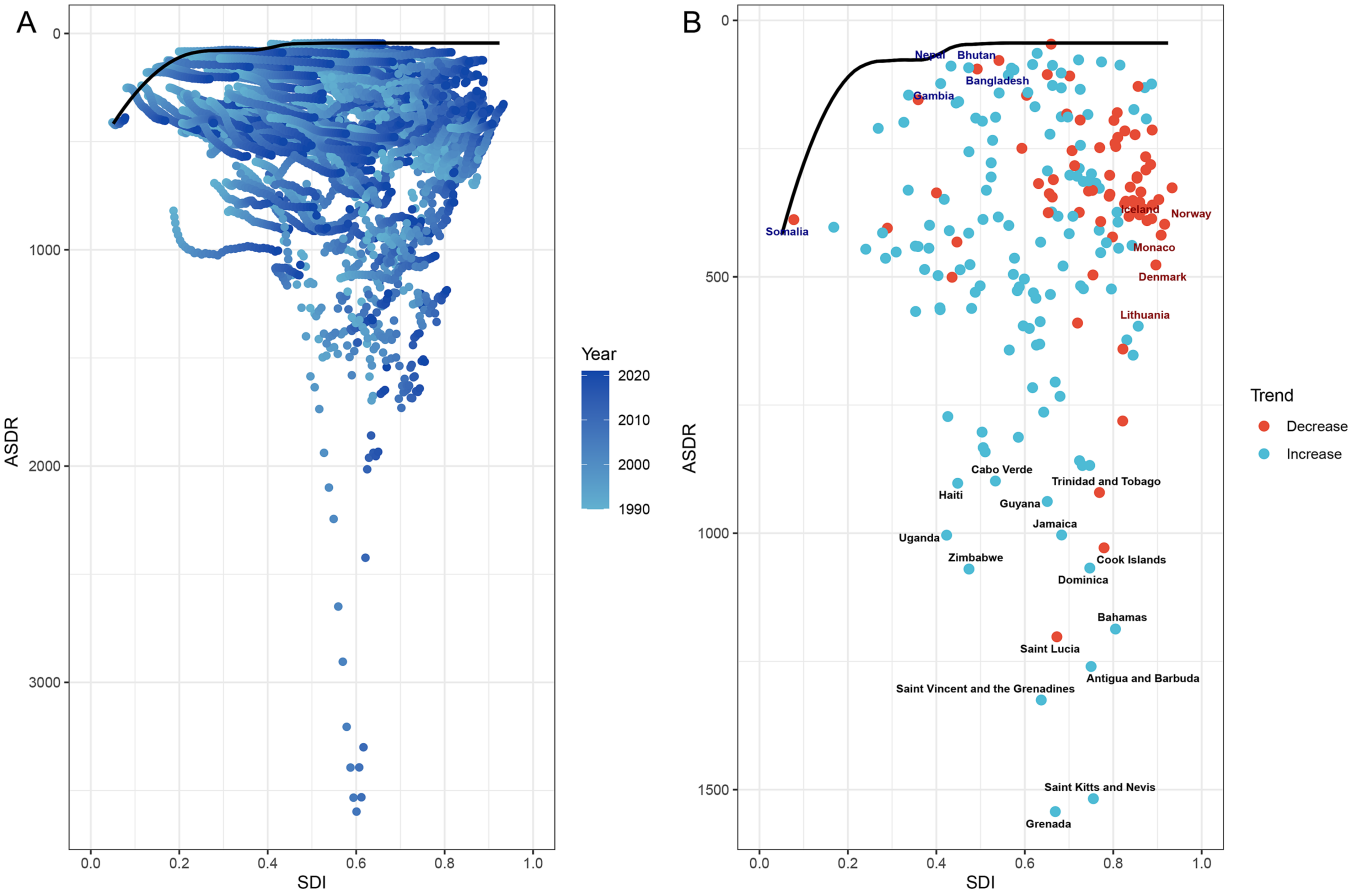
Frontier analysis of PCa-related ASDR and SDI trajectories across 204 countries and territories from 1990 to 2021. **(A)** Temporal progression of ASDR-SDI relationships, with gradient shading (light to dark blue) denoting years from 1990 to 2021. **(B)** Efficiency frontier (solid black line) comparing ASDR and SDI trends over time. Red dots represent countries with reduced ASDR from 1990 to 2021; blue dots denote increased ASDR for PCa from 1990 to 2021. Highlighted labels include: top five low-SDI countries with minimal effective difference from the frontier (blue), high-SDI countries with substantial effective difference (red), and the top 15 countries exhibiting the largest effective difference (black). PCa, prostate cancer; SDI, socio-demographic index; ASDR, age-standardized disability-adjusted life years.

Frontier analysis of PCa DALYs revealed substantial heterogeneity in unmet health potential across 204 countries and territories stratified by socio-demographic development levels between 1990 and 2021 (Figure 10A). Countries with low SDI (<0.60), such as Somalia, Nepal, Gambia, Bangladesh, and Bhutan, were clustered near the efficiency frontier (solid black line) (Figure 10B; Supplementary Table S6). These nations exhibited minimal effective differences (depicted in blue), reflecting near-optimal health outcomes relative to their development context. Conversely, high SDI countries (>0.80), including Norway, Iceland, Monaco, Denmark, and Lithuania (depicted in red), showed the largest effective differences due to their substantial deviation from the frontier. Notably, the top 15 countries with the most pronounced effective differences (depicted in black), such as Grenada, Haiti, Uganda, and Zimbabwe, were predominantly concentrated in Low-middle, Middle, and High-middle SDI regions (Figure 10B; Supplementary Table S6). These findings suggested that high-SDI countries and territories, despite advanced development, had the greatest unrealized potential for health efficiency improvements.

## 4 Discussion

Based on the GBD 2021 database, this study comprehensively evaluated the disease burden of PCa at the global, regional, and national levels stratified by age and SDI from 1990 to 2021. Our findings reveal a sustained global increase in PCa incidence, prevalence, DALYs, and mortality over the past three decades, with marked disparities across geographic regions and socioeconomic strata. Disease burden exhibited a strong correlation with aging populations and socioeconomic development. HICs demonstrated elevated ASIR and ASPR, coupled with declining ASDR and ASMR. Conversely, LMICs experienced upward trends in ASDR and ASMR, underscoring systemic inequities in healthcare access and infrastructure. These results highlight the critical role of socioeconomic determinants in shaping PCa outcomes. By providing stratified evidence across development contexts, this analysis offers actionable insights to optimize diagnosis, treatment, and prognosis through resource-sensitive strategies. Addressing these imbalances is essential to mitigate the escalating global health challenge posed by PCa.

The global burden of PCa has increased substantially over the past three decades, with incidence rates rising approximately 1.6-fold and mortality rates nearly 2.0-fold worldwide. Notably, while high SDI regions, including high-income North America and Australasia, demonstrated persistently elevated ASIR and ASPR, these regions concurrently exhibited lower MIRs, indicative of comparatively higher survival rates. This pattern aligned with prior studies (30, 31), and might be attributed to the easy accessibility of PCa screening, advanced therapeutic interventions, and robust survivorship care initiatives (1). Conversely, LMICs experienced significant upward trends in ASIR and ASPR between 1990 and 2021, likely attributed to incremental improvements in PSA-based screening availability and diagnostic practices during this period (32, 33). Despite rising numbers of DALYs and mortality cases worldwide, the ASDR and ASMR substantially decreased in regions in middle to high SDI regions by 2021. These reductions mirrored epidemiological observations in HICs, where sustained cancer control programs, including population-level screenings, dietary modifications, and lifestyle interventions, have contributed to stabilized or declining PCa mortality trajectories (34, 35). Conversely, LMICs witnessed substantial increases in ASDR and ASMR during the same timeframe. These disparities likely stem from systemic challenges in healthcare infrastructure, including limited implementation of early detection protocols, delayed diagnoses, and suboptimal treatment accessibility. Compounding these issues are regionally prevalent risk factors such as tobacco use and insufficient public health prioritization of PCa prevention, collectively exacerbating late-stage presentation and poorer clinical outcomes (3, 36, 37).

This study demonstrated that the incidence, prevalence, DALYs, and mortality rates of PCa correlated strongly with advanced age, exhibiting pronounced acceleration in males aged over 50 years. These findings aligned with existing literature underscoring the elevated risk of PCa among older adult populations, compounded by frequent comorbidities such as cardiovascular disease, diabetes, and osteoporosis. Such multimorbidity complicates both disease management and treatment tolerability, often influencing clinical decisions to defer aggressive intervention (e.g., surgery, radiation therapy) (38–40). Notably, our study identified the highest incidence, DALYs, and mortality rates in the 85+ age cohort related to the younger group. Given the frequent diagnosis of advanced-stage PCa in older adult men, active surveillance and palliative care emerge as pivotal strategies to optimize prognosis and minimize overtreatment (41). Concurrently, studies emphasize that tailoring therapeutic regimens to individual functional status, comorbidities, and patient preferences significantly improves quality of life outcomes in this demographic (42, 43). Moreover, our findings indicated that aging represented the foremost contributor to PCa-related DALYs globally, surpassing population growth as a determinant of disease burden. This underscores the urgency of prioritizing regions with aging populations in public health planning. In our study, Low-middle SDI quintile, such as Southern Sub-Saharan Africa, Western Sub-Saharan Africa, and Caribbean ranked as the top three regions in DALYs cases of PCa. Despite persistent population growth in these regions, declining fertility rates and youth cohorts have precipitated a paradoxical demographic shift toward aging populations in recent decades (44). This trend poses multifaceted challenges, including inadequate healthcare infrastructure, limited adoption of advanced medical technologies, and inequitable resource allocation for geriatric care (45, 46). Collectively, these systemic gaps exacerbate delays in diagnosis, hinder access to guideline-concordant treatments, and amplify PCa burden in resource-constrained settings.

A previous study using the GBD2019 database reported that Middle SDI quintiles displayed the highest EAPC for ASIR of PCa, concurrently exhibiting elevated EAPCs for ASDR and ASMR despite their comparatively low ASIR trajectories (3). The updated findings from GBD 2021 database, however, demonstrated a shifting epidemiological landscape: regions with Low-middle SDI, such as North Africa and Middle East, East Asia, and Eastern Europe, exhibited the most rapid escalation in ASIR, ASPR, ASDR, and ASMR of PCa. This trend suggests gradual advancements in PCa screening accessibility within these emerging regions, albeit persistent systemic barriers to implementing timely, guideline-adherent treatments that might mitigate mortality (47, 48). In contrast, High-SDI regions such as High-income North America, Australasia, and Western Europe demonstrated stabilized or declining EAPCs for ASIR, ASPR, ASDR, and ASMR, which was also corroborated by earlier research (15). Accumulating evidence attributes the declining PCa incidence in HICs to reduced reliance on PSA screening, alongside the integration of multiparametric magnetic resonance imaging into diagnostic protocols. Such advancements enable more precise identification of clinically significant PCa, thereby curbing overdiagnosis and unnecessary biopsies (32, 49). Moreover, studies highlight the association between lower PCa-related DALYs and mortality rates in high SDI regions with superior quality in healthcare delivery, including early detection programs, prevention interventions, and optimized therapeutic management (50).

Despite persistent disparities in the EAPCs of ASIR, ASPR, ASDR, and ASMR across different SDI quintiles over the past 30 years, positive associations were found between the elevated ASIR/ASPR and high SDI regions. This observation aligned with prior studies documenting higher ASIR of PCa in nations with advanced healthcare systems and socioeconomic development (3). Specifically, high SDI regions such as America, Australia, and Germany have achieved substantial advancements in implementing nationwide screening programs utilizing novel biomarkers and radiological imaging, facilitating early detection even in clinically indolent cases (51). Notably, demographic shifts in these regions, including aging populations, prolonged life expectancy, and declining fertility rates, likely further amplify ASIR and ASPR by increasing cumulative exposure to oncogenic risk factors (52). Conversely, our findings demonstrate an inverse association between SDI levels and EAPCs of ASDR/ASMR globally. PCa patients in low SDI regions disproportionately endure elevated mortality burdens and diminished quality of life, a disparity attributable to restricted access to quality healthcare, limited health literacy, financial constraints to treatment, and systemic inequalities (53, 54). These structural disparities necessitate holistic interventions targeting policy reform, equitable resource allocation, and community-centered education to mitigate preventable PCa morbidity and mortality.

Notably, the cases of DALYs for PCa doubled in 2021 compared to 1990, with the steepest increase concentrated in Low-middle and Middle SDI regions, including Southeast Asia, North Africa and Middle East, and High-income Asia Pacific. In High-income Asia Pacific countries such as Japan, South Korea, and Singapore, rapid industrialization and advancements in healthcare have accelerated demographic aging, elevating DALYs through increased prevalence of age-related chronic conditions among older adult populations (45). Frontier analysis further highlighted the inequities in DALYs across different countries and regions. For instance, low-income countries such as Somalia, Gambia, and Nepal—despite constrained healthcare infrastructures—have achieved substantial health gains in PCa-specific life expectancy, providing useful insights for reducing disease burden in resource-limited settings. Conversely, middle to high SDI countries, such as Grenada, Bahamas, Iceland, Norway, and Denmark, lag behind the efficiency frontier despite ostensibly adequate national-level performances, suggesting that these countries have significant opportunities to enhance healthcare efficiency and outcomes to mitigate DALYs associated with PCa (55). Therefore, these findings provide valuable insights into health system efficiency, pinpointing regions where interventions could maximally reduce PCa burden. By benchmarking optimal health outcomes achievable under prevailing socioeconomic constraints, researchers can guide policymakers in identifying barriers to progress and prioritizing context-specific reform (35, 56). Proposed strategies include scaling preventive care initiatives, investing in diagnostic and therapeutic infrastructure, and implementing workforce capacity-building programs tailored to regional epidemiologic profiles (57).

The current study has several limitations. Although our analysis reveals a pronounced near-doubling of PCa incidence over the 30-year period, these trends must be interpreted cautiously due to methodological constraints, heterogeneous data quality, and potential biases that may amplify or underestimate true epidemiological patterns. First, the adoption of PSA testing as a diagnostic tool introduces significant variability in reported incidence rates. PSA screening practices differ markedly across regions and over time, creating temporal and geographic biases between HICs and LMICs. For instance, limited access to screening infrastructure in LMICs likely obscured the true epidemiological burden in earlier decades. This temporal mismatch in data availability—where some regions contributed robust data only in recent years—may exaggerate the perceived “global” doubling of cases, as earlier estimates from LMICs often relied on extrapolation. Second, the GBD 2021 employs statistical modeling to address gaps in countries with sparse or absent cancer registries. While indispensable for generating global estimates, these models inherently propagate uncertainties. Furthermore, shifts in GBD modeling assumptions or input data sources over the three-decade period may introduce unquantified variability into longitudinal trend analyses. Third, disparities in health infrastructure and data quality critically affect reliability. Many LMICs lack systematic cancer surveillance systems, leading to underreporting of incidence and mortality. This undermines the granularity of estimates and complicates cross-country comparisons. Fourth, DALYs as a metric may insufficiently capture sociocultural determinants of health-seeking behavior and disease perception. For example, stigma surrounding PCa in some populations may suppress healthcare utilization, artificially deflating DALY estimates, while increased health awareness in affluent regions could inflate reported burdens. To address these limitations, further research must adopt multidisciplinary approaches integrating genetic profiling, environmental exposure history, longitudinal lifestyle trajectories, and health-system performance to elucidate the true drivers of PCa epidemiology.

## 5 Conclusion

The burden of PCa remains a significant public health concern globally. Although the ASIR, ASPR, ASDR, and ASMR have declined or stabilized worldwide over the past three decades, the crude cases of incidence, prevalence, DALYs, and mortality persistently increased between 1990 and 2021. These trends exhibited pronounced disparities across age strata, sociodemographic development tiers, and geographical regions. Notably, lower SDI regions displayed disproportionately adverse epidemiological trajectories, whereas higher SDI settings continued to face gaps in attainable health outcomes despite advanced healthcare infrastructure. These findings highlight the critical need for equity-driven policymaking, targeted resource allocation, and health system optimization to enhance the cost-effectiveness of interventions aimed at reducing the global disease burden of PCa.

## Data Availability

The original contributions presented in the study are included in the article/Supplementary material, further inquiries can be directed to the corresponding author.

## Footnotes

^1^ https://ghdx.healthdata.org/gbd-2021/sources
