# Supplementary material for: The global, regional, and national prostate cancer burden and trends from 1990 to 2021, results from the global burden of disease study 2021
Source: Front Public Health. 2025 May 21;13:1553747. doi: 10.3389/fpubh.2025.1553747 (PMC12133484; doi:10.3389/fpubh.2025.1553747)
Supplement: Supplementary file 1 [file Data_Sheet_1.docx]

***Supplementary materials***

**Table of content**

Figure S1. Global distribution of prevalence burden for PCa in 204 countries and territories2

Figure S2. Global distribution of DALYs burden for PCa in 204 countries and territories3

Figure S3. Global distribution of mortality burden for PCa in 204 countries and territories4

Table S1. The global prevalence of PCa and its estimated trends from 1990 to 20215

Table S2. The global DALYs of PCa and its estimated trends from 1990 to 20217

Table S3. The global mortality of PCa and its estimated trends from 1990 to 20219

Table S4. Mortality-to-incidence ratio among PCa patients from 1990 to 2021.11

Table S5. Changes of DALYs number for PCa due to population-level determinants worldwide and in SDI quintiles from 1990 to 202113

Table S6. Frontier DALYs and effective difference by 204 countries and territories in 202114

**Supplementary Figures**


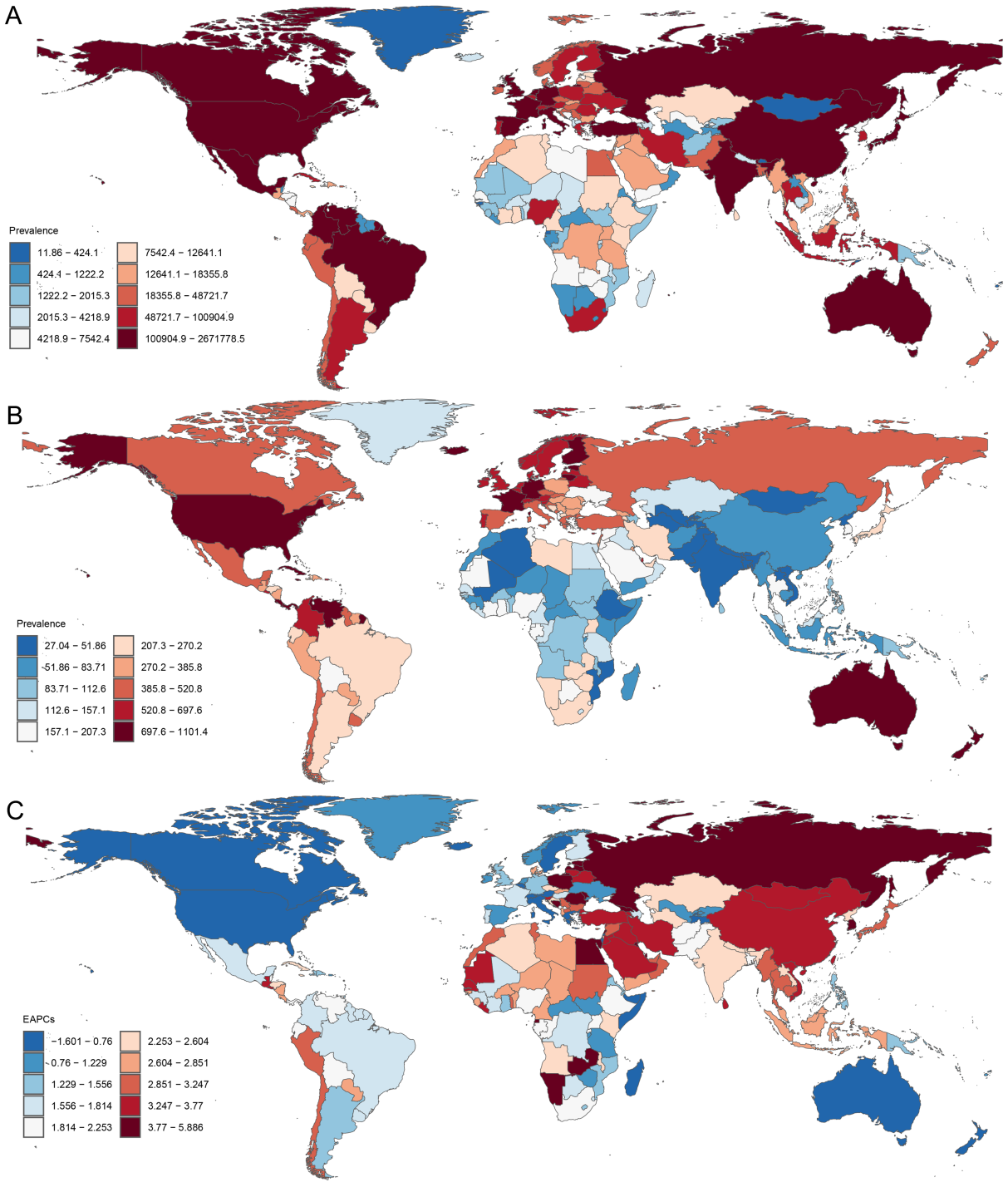


**Figure S1.** Global distribution of prevalence burden for PCa in 204 countries and territories. (A) The prevalence of PCa in 2021; (B) ASPR of PCa in 2021; (C) EAPC of ASPR of PCa from 1990 to 2021. ASPR, age-standardized prevalence; EAPC, estimated annual percentage change; PCa, prostate cancer.


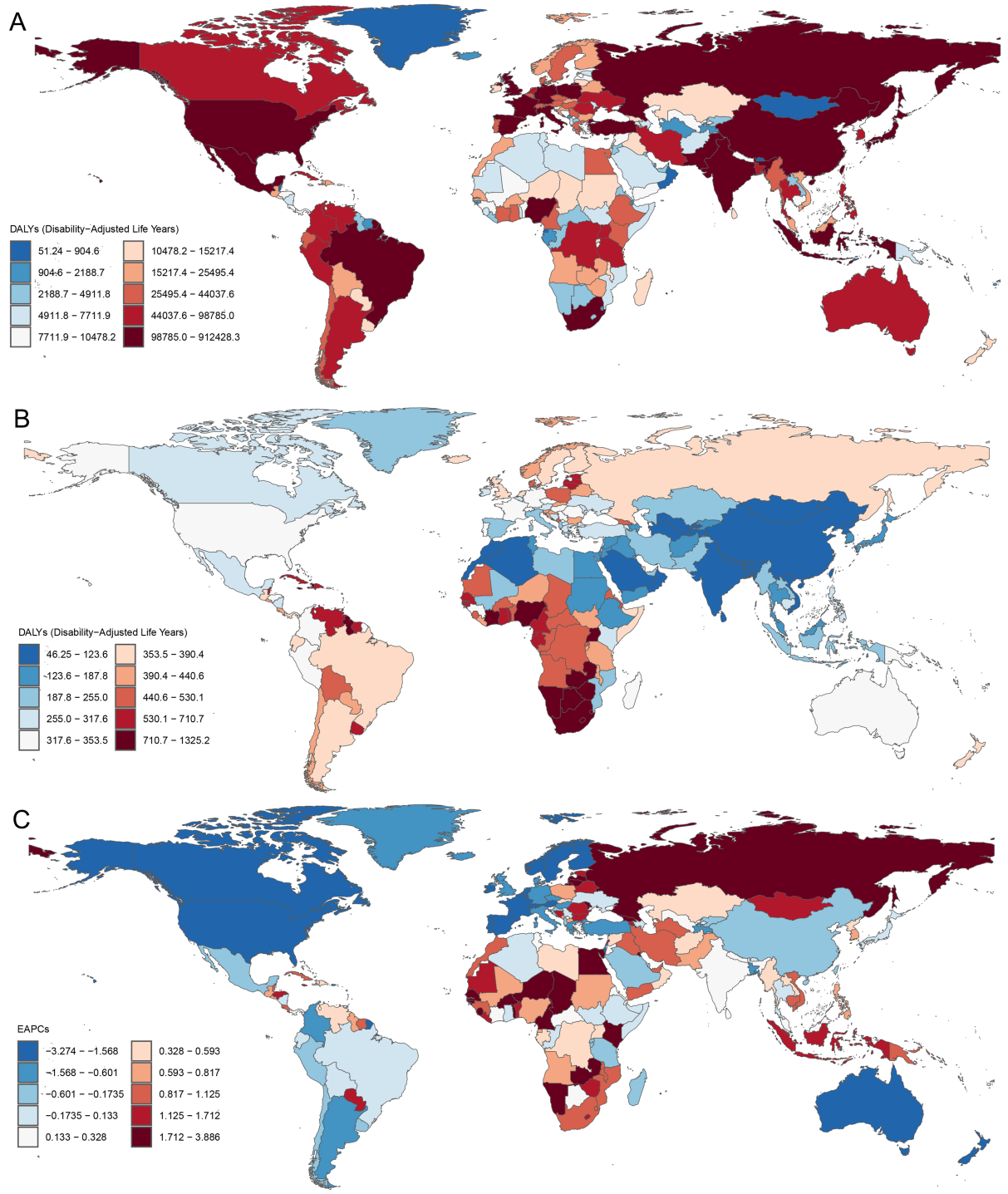


**Figure S2.** Global distribution of DALYs burden for PCa in 204 countries and territories. (A) The DALYs of PCa in 2021; (B) ASDR of PCa in 2021; (C) EAPC of ASDR of PCa from 1990 to 2021. ASDR, age-standardized disability-adjusted life years; DALYs, disability-adjusted life years; EAPC, estimated annual percentage change; PCa, prostate cancer.


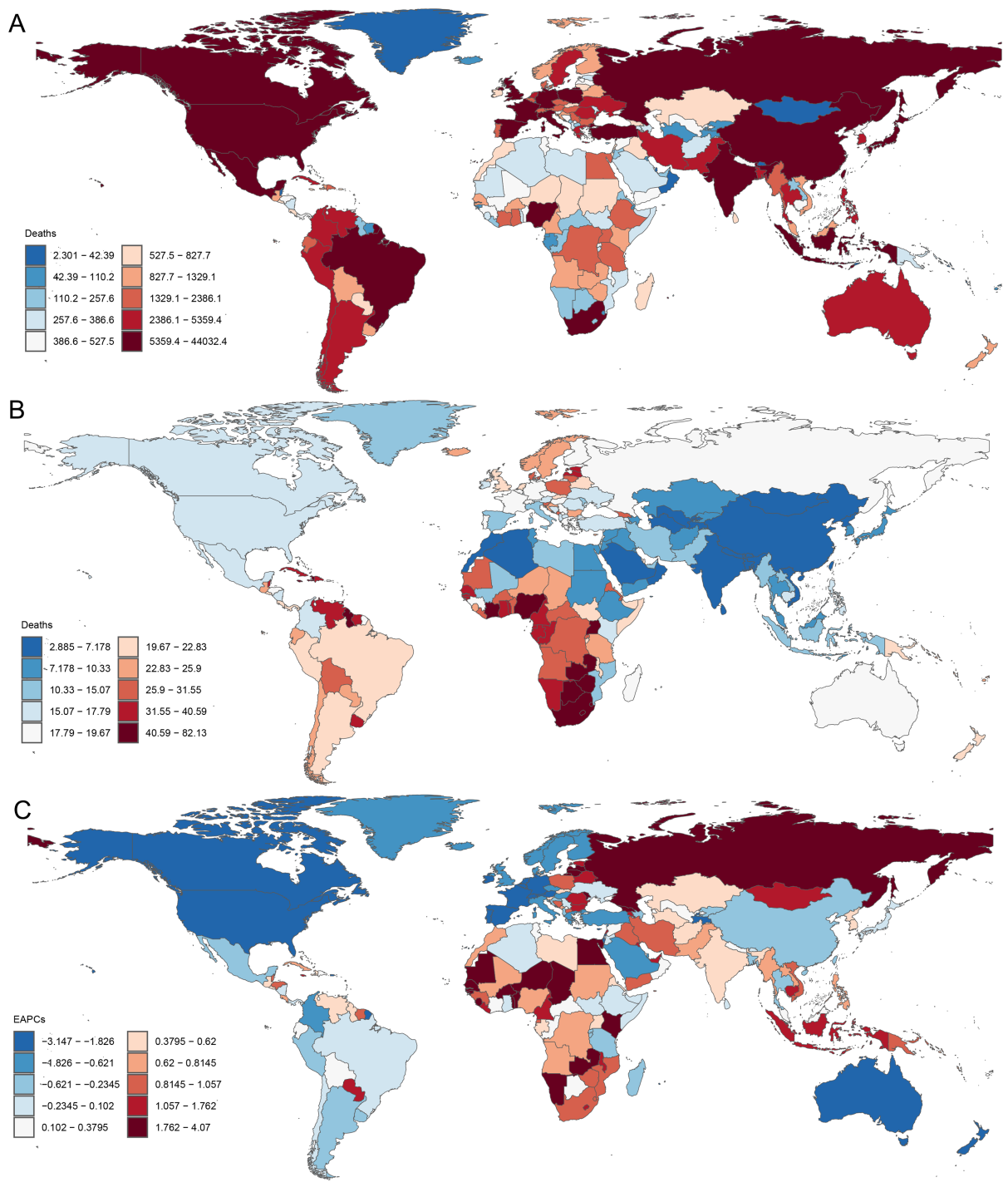


**Figure S3.** Global distribution of mortality burden for PCa in 204 countries and territories. (A) The mortality of PCa in 2021; (B) ASMR of PCa in 2021; (C) EAPC of ASMR of PCa from 1990 to 2021. ASMR, age-standardized mortality; EAPC, estimated annual percentage change; PCa, prostate cancer.

**Table S1.** The global prevalence of PCa and its estimated trends from 1990 to 2021.

| **Characteristics** | **1990** | | **2021** | | **1990-2021** | **1990-2021** |
| --- | --- | --- | --- | --- | --- | --- |
|  | **Prevalence cases**  **No. (95% UI)** | **ASPR per 100,000**  **No. (95% UI)** | **Prevalence cases**  **No. (95% UI)** | **ASPR per 100,000**  **No. (95% UI)** | **Cases change**  **(95% UI)** | **EAPC**  **(95% CI)** |
| Global | 3596219.90(3445436.84,3705436.00) | 218.33(208.48,225.67) | 10387520.53(9705680.48,10904399.86) | 260.05(243.39,272.68) | 188.85(176.52,200.98) | 0.42(0.26,0.58) |
| High SDI | 2607410.10(2513878.14,2683485.45) | 555.13(533.78,572.10) | 5987871.58(5660940.43,6245000.12) | 612.57(579.53,638.50) | 129.65(119.30,138.75) | 0.14(-0.04,0.33) |
| High-middle SDI | 45356.69(31012.02,56284.30) | 141.14(134.08,147.86) | 163701.17(108548.35,202253.54) | 225.91(202.96,244.08) | 261.44(232.14,291.74) | 1.52(1.31,1.72) |
| Middle SDI | 104283.94(85175.12,116315.28) | 59.33(52.79,63.63) | 570720.71(479427.98,656395.14) | 125.63(108.85,140.62) | 499.91(448.46,559.50) | 2.24(2.03,2.45) |
| Low-middle SDI | 265209.17(233035.57,285412.34) | 36.99(30.58,41.20) | 1591026.12(1374887.42,1786559.34) | 85.84(72.80,98.20) | 447.28(377.18,528.53) | 2.66(2.51,2.80) |
| Low SDI | 570033.59(540995.49,596895.81) | 43.11(29.89,53.04) | 2060335.15(1849680.86,2229490.22) | 70.35(47.20,86.16) | 260.92(212.27,326.54) | 1.56(1.46,1.66) |
| Andean Latin America | 10503.92(8360.14,13159.54) | 113.95(91.17,142.61) | 75651.85(55386.14,104359.85) | 275.22(201.70,378.08) | 620.22(445.32,826.55) | 2.76(2.53,3.00) |
| Australasia | 85970.47(79053.74,91672.67) | 822.04(757.11,873.29) | 235141.09(204068.06,271001.38) | 906.45(787.27,1045.55) | 173.51(137.14,221.52) | -0.10(-0.78,0.59) |
| Caribbean | 45217.82(41832.85,49265.00) | 365.05(338.20,397.30) | 163762.06(137670.09,189249.65) | 653.29(550.36,754.63) | 262.16(204.97,327.04) | 1.77(1.56,1.99) |
| Central Asia | 10957.52(10255.67,11668.38) | 60.49(56.39,64.36) | 31229.79(28034.14,34704.24) | 89.92(81.23,99.58) | 185.01(153.97,219.31) | 2.10(1.75,2.45) |
| Central Europe | 83362.16(78626.66,88280.59) | 131.52(123.97,139.33) | 338084.13(306991.19,370396.68) | 338.01(306.97,369.88) | 305.56(269.29,346.91) | 3.27(2.99,3.55) |
| Central Latin America | 87911.21(83465.64,91295.29) | 233.01(221.38,242.04) | 558238.59(479996.26,640212.82) | 492.55(424.50,563.85) | 535.00(440.40,626.24) | 1.94(1.56,2.32) |
| Central Sub-Saharan Africa | 5016.99(3284.49,6624.37) | 55.74(37.28,72.10) | 20513.54(12800.71,27624.96) | 95.70(60.18,128.31) | 308.88(204.60,448.89) | 1.79(1.55,2.03) |
| East Asia | 79696.33(60406.43,97784.10) | 20.67(16.05,25.21) | 692463.58(512654.86,915807.24) | 64.31(47.85,84.41) | 768.88(551.91,1024.66) | 3.75(3.57,3.92) |
| Eastern Europe | 147970.59(140097.02,155337.51) | 152.36(144.26,159.90) | 571269.56(512389.56,625712.21) | 411.07(370.69,448.37) | 286.07(245.70,326.47) | 3.73(3.44,4.01) |
| Eastern Sub-Saharan Africa | 19182.41(12088.61,24213.71) | 55.39(35.67,69.15) | 70409.64(45743.83,90459.26) | 93.37(62.31,118.20) | 267.05(200.04,372.55) | 1.68(1.58,1.78) |
| High-income Asia Pacific | 79023.29(74683.31,83291.66) | 102.46(96.51,107.92) | 534446.77(475300.22,586961.77) | 237.31(211.16,260.35) | 576.32(505.84,643.90) | 3.02(2.45,3.58) |
| High-income North America | 1608512.07(1545487.55,1663298.54) | 1051.65(1008.89,1088.32) | 2829392.88(2698232.77,2946882.97) | 910.62(867.85,948.35) | 75.90(68.10,84.06) | -0.86(-0.98,-0.73) |
| North Africa and Middle East | 52612.12(39259.82,62722.66) | 65.76(50.51,78.79) | 432154.78(310010.29,524900.01) | 200.42(144.79,242.38) | 721.40(595.36,875.43) | 3.86(3.73,4.00) |
| Oceania | 909.18(648.33,1212.66) | 70.87(51.85,92.07) | 3395.42(2209.05,4627.25) | 101.39(67.82,137.57) | 273.46(198.23,395.66) | 1.21(1.16,1.25) |
| South Asia | 46419.34(33953.45,55552.01) | 16.93(12.54,20.17) | 262891.39(213983.75,350658.83) | 36.99(30.23,49.02) | 466.34(343.63,642.05) | 2.31(2.16,2.45) |
| Southeast Asia | 42332.15(30155.45,48703.58) | 39.33(28.39,45.11) | 274478.29(182878.31,336698.44) | 95.91(64.65,117.10) | 548.39(427.97,673.53) | 2.92(2.86,2.98) |
| Southern Latin America | 34782.93(31405.73,38534.83) | 171.81(155.94,190.03) | 118558.38(101797.25,136410.46) | 303.87(261.43,348.22) | 240.85(186.88,295.67) | 1.83(1.41,2.24) |
| Southern Sub-Saharan Africa | 15223.44(10869.52,20565.25) | 142.03(102.76,187.81) | 58561.90(44816.21,70006.40) | 251.77(193.09,298.10) | 284.68(205.22,412.84) | 1.99(1.91,2.06) |
| Tropical Latin America | 58446.83(55455.27,61316.55) | 143.59(136.04,150.57) | 311471.24(292602.12,329482.39) | 270.67(254.10,286.45) | 432.91(396.31,472.34) | 1.71(1.30,2.13) |
| Western Europe | 1047293.61(1005897.48,1095046.40) | 427.11(409.99,446.13) | 2670002.49(2459710.19,2848675.32) | 625.03(577.47,667.95) | 154.94(135.85,173.95) | 1.13(0.73,1.54) |
| Western Sub-Saharan Africa | 34875.53(20320.63,45889.25) | 86.70(51.95,112.20) | 135403.16(71247.50,184783.43) | 159.95(86.08,215.29) | 288.25(191.11,419.63) | 2.13(2.03,2.22) |

**Notes.** Abbreviations: ASPR, age-standardized prevalence; CI, confidence interval; PC, percentage change; EAPC, estimated annual percentage change; UI, uncertainty interval; PCa, prostate cancer

**Table S2.** The global DALYs of PCa and its estimated trends from 1990 to 2021.

| **Characteristics** | **1990** | | **2021** | | **1990-2021** | **1990-2021** |
| --- | --- | --- | --- | --- | --- | --- |
|  | **DALYs cases**  **No. (95% UI)** | **ASDR per 100,000**  **No. (95% UI)** | **DALYs cases**  **No. (95% UI)** | **ASDR per 100,000**  **No. (95% UI)** | **Cases change**  **(95% UI)** | **EAPC**  **(95% CI)** |
| Global | 4146718.76(3754315.11,4401619.73) | 275.30(251.66,292.14) | 8141561.07(7177066.89,8809184.40) | 217.83(192.65,235.53) | 96.34(86.16,107.71) | -0.96(-1.05,-0.87) |
| High SDI | 1997193.66(1898545.95,2080261.00) | 449.81(425.57,468.55) | 2788077.76(2562127.53,2985290.67) | 280.72(257.96,300.71) | 39.60(32.73,45.28) | -1.83(-1.93,-1.73) |
| High-middle SDI | 863489.75(802546.25,909741.07) | 239.93(223.81,253.02) | 1691418.00(1500583.11,1855683.41) | 201.01(179.17,220.19) | 95.88(81.17,111.43) | -0.73(-0.89,-0.57) |
| Middle SDI | 628475.58(513148.28,706515.14) | 164.03(137.01,186.89) | 1907052.07(1575666.47,2199147.58) | 168.77(139.74,195.09) | 203.44(175.80,241.77) | -0.08(-0.25,0.10) |
| Low-middle SDI | 392954.01(294151.27,460547.59) | 152.92(116.25,179.89) | 1135076.28(909522.52,1341822.95) | 189.22(153.60,223.29) | 188.86(146.36,239.59) | 0.62(0.50,0.73) |
| Low SDI | 257784.67(167408.12,328065.24) | 268.41(175.52,339.96) | 605967.23(385983.93,754880.92) | 294.26(189.52,363.32) | 135.07(103.13,182.78) | 0.24(0.19,0.30) |
| Andean Latin America | 32665.44(26343.32,40676.94) | 375.70(305.29,465.39) | 98256.83(72453.49,131978.04) | 372.49(275.16,500.95) | 200.80(135.55,278.76) | -0.11(-0.25,0.04) |
| Australasia | 70604.67(64290.39,74808.06) | 713.17(649.53,757.07) | 93927.97(79391.04,109091.32) | 354.41(299.70,411.98) | 33.03(15.06,57.77) | -2.67(-3.07,-2.27) |
| Caribbean | 69188.34(63927.95,78173.27) | 591.66(545.96,667.76) | 167719.11(143254.16,196782.50) | 680.84(582.10,799.04) | 142.41(109.83,180.55) | 0.36(0.20,0.52) |
| Central Asia | 28922.95(27022.25,30717.67) | 168.80(157.68,179.29) | 53861.81(48667.59,59742.58) | 174.31(158.35,191.95) | 86.23(68.05,106.63) | 0.79(0.54,1.03) |
| Central Europe | 183437.52(174215.76,193955.29) | 311.60(295.69,330.47) | 365176.14(331181.49,395938.67) | 380.69(345.46,412.55) | 99.07(83.23,116.44) | 0.49(0.31,0.68) |
| Central Latin America | 119032.51(113700.62,123742.24) | 339.05(322.40,352.97) | 386729.29(334403.87,442802.01) | 357.67(310.25,408.84) | 224.89(181.76,269.59) | -0.22(-0.44,0.01) |
| Central Sub-Saharan Africa | 30972.27(19517.06,40954.73) | 388.44(253.69,522.15) | 80368.73(48683.14,109548.03) | 462.63(277.39,637.06) | 159.49(95.12,248.40) | 0.57(0.44,0.70) |
| East Asia | 235719.22(174787.91,299480.64) | 76.32(57.80,97.78) | 729148.48(552838.50,984062.49) | 79.68(60.09,105.85) | 209.33(125.76,305.93) | -0.20(-0.40,-0.00) |
| Eastern Europe | 215265.30(203043.24,225894.48) | 240.07(226.23,251.93) | 482141.63(429316.03,539566.38) | 370.67(332.05,413.25) | 123.98(98.54,150.52) | 1.51(1.41,1.60) |
| Eastern Sub-Saharan Africa | 109426.49(65469.50,139655.77) | 342.80(206.98,435.99) | 250292.60(159631.36,324270.14) | 371.75(238.72,471.99) | 128.73(88.15,193.47) | 0.18(0.15,0.21) |
| High-income Asia Pacific | 90487.35(85908.83,95678.11) | 119.10(112.14,125.97) | 299881.67(267886.81,324754.39) | 128.79(115.29,139.46) | 231.41(199.54,254.56) | 0.00(-0.29,0.29) |
| High-income North America | 849664.13(800772.90,899662.12) | 574.24(540.37,607.97) | 1005025.19(921818.41,1086607.63) | 327.02(300.14,353.70) | 18.28(12.92,24.24) | -2.25(-2.40,-2.09) |
| North Africa and Middle East | 101947.47(76144.12,126136.57) | 149.44(111.74,189.31) | 327766.18(230650.66,392311.48) | 173.58(123.07,209.38) | 221.50(168.82,288.83) | 0.51(0.44,0.59) |
| Oceania | 3464.55(2362.83,4821.50) | 318.69(218.47,438.25) | 10441.03(6515.12,14641.27) | 361.58(229.77,512.13) | 201.37(132.78,311.22) | 0.53(0.48,0.59) |
| South Asia | 230783.74(162767.36,283270.80) | 92.25(65.02,113.26) | 665394.63(540224.94,911799.89) | 104.26(85.19,143.01) | 188.32(119.99,283.29) | 0.08(-0.06,0.22) |
| Southeast Asia | 144347.80(99444.22,169252.58) | 148.08(102.87,174.39) | 491686.02(324530.78,600823.49) | 190.74(125.11,231.98) | 240.63(178.37,311.37) | 0.81(0.74,0.89) |
| Southern Latin America | 88207.83(79474.01,98160.03) | 462.30(418.14,514.59) | 148542.01(127818.53,170368.31) | 394.12(340.04,452.15) | 68.40(42.24,94.81) | -0.55(-0.88,-0.21) |
| Southern Sub-Saharan Africa | 58464.16(42251.65,77995.90) | 598.70(437.40,794.34) | 155390.89(115234.52,184542.13) | 774.40(563.55,905.89) | 165.79(107.22,253.81) | 0.94(0.72,1.15) |
| Tropical Latin America | 137348.59(130432.24,143185.34) | 374.99(352.89,392.02) | 405586.31(378810.97,431067.69) | 376.24(349.02,400.47) | 195.30(178.45,213.17) | -0.11(-0.45,0.24) |
| Western Europe | 1157156.81(1104105.69,1204270.56) | 495.12(470.67,515.82) | 1443478.22(1291508.08,1568625.67) | 319.95(287.64,347.77) | 24.74(15.48,34.07) | -1.68(-1.82,-1.54) |
| Western Sub-Saharan Africa | 189611.61(108491.15,250811.63) | 520.35(302.27,680.53) | 480746.35(252059.02,651050.56) | 646.45(343.40,860.10) | 153.54(93.71,240.04) | 0.80(0.75,0.84) |

**Notes.** Abbreviations: ASDR, age-standardized disability-adjusted life years; CI, confidence interval; PC, percentage change; EAPC, estimated annual percentage change; UI, uncertainty interval; PCa, prostatic cancer

**Table S3.** The global mortality of PCa and its estimated trends from 1990 to 2021.

| **Characteristics** | **1990** | | **2021** | | **1990-2021** | **1990-2021** |
| --- | --- | --- | --- | --- | --- | --- |
|  | **Mortality cases**  **No. (95% UI)** | **ASMR per 100,000**  **No. (95% UI)** | **Mortality cases**  **No. (95% UI)** | **ASMR per 100,000**  **No. (95% UI)** | **Cases change**  **(95% UI)** | **EAPC**  **(95% CI)** |
| Global | 211971.54(194221.85,224327.73) | 16.35(15.02,17.28) | 432463.33(381872.79,463645.28) | 12.63(11.16,13.55) | 104.02(92.73,116.11) | -0.58(-0.73,-0.44) |
| High SDI | 105148.97(99286.29,108362.97) | 25.71(24.06,26.61) | 154422.87(138844.11,163654.78) | 15.35(13.80,16.27) | 46.86(37.88,53.25) | -1.35(-1.57,-1.13) |
| High-middle SDI | 43451.66(40561.82,45902.15) | 14.25(13.20,15.09) | 90322.92(79773.36,98853.75) | 11.73(10.33,12.86) | 107.87(90.44,124.72) | -0.21(-0.41,-0.00) |
| Middle SDI | 31439.16(26222.28,35879.52) | 10.14(8.57,11.64) | 99769.49(82565.02,115535.27) | 10.03(8.30,11.60) | 217.34(186.57,258.50) | 0.21(0.05,0.36) |
| Low-middle SDI | 19447.79(14790.69,22960.71) | 8.77(6.78,10.39) | 57755.76(46941.22,68428.62) | 10.92(9.02,12.93) | 196.98(152.90,247.90) | 0.88(0.78,0.98) |
| Low SDI | 12124.00(7907.02,15318.90) | 15.02(9.92,19.00) | 29431.23(18997.65,36227.13) | 16.80(10.86,20.59) | 142.75(111.45,188.17) | 0.30(0.26,0.34) |
| Andean Latin America | 1839.39(1502.85,2258.00) | 22.69(18.80,27.69) | 5631.80(4181.03,7475.31) | 22.26(16.58,29.52) | 206.18(144.80,284.07) | 0.29(0.14,0.43) |
| Australasia | 3640.77(3316.57,3842.57) | 40.72(36.91,43.16) | 5314.15(4490.64,6098.73) | 19.76(16.71,22.70) | 45.96(24.20,71.83) | -1.36(-1.82,-0.89) |
| Caribbean | 3778.86(3479.23,4244.80) | 34.83(32.02,39.05) | 9257.64(7967.22,10783.23) | 38.35(32.98,44.63) | 144.99(113.87,181.75) | 0.54(0.41,0.68) |
| Central Asia | 1267.59(1179.99,1345.66) | 8.42(7.84,8.97) | 2405.18(2182.71,2651.64) | 9.12(8.32,10.01) | 89.74(72.55,109.99) | 0.52(0.29,0.74) |
| Central Europe | 9399.07(8917.84,10003.36) | 17.68(16.69,18.84) | 19779.46(18084.22,21356.02) | 21.49(19.56,23.19) | 110.44(93.45,128.16) | 0.76(0.61,0.91) |
| Central Latin America | 6188.39(5850.64,6433.84) | 19.31(18.10,20.11) | 20037.41(17440.11,22605.95) | 19.52(17.03,21.95) | 223.79(186.09,263.60) | 0.25(0.04,0.45) |
| Central Sub-Saharan Africa | 1448.52(939.28,1937.33) | 22.86(14.74,31.44) | 3722.13(2245.87,5109.91) | 27.70(16.75,38.72) | 156.96(96.11,244.04) | 0.51(0.43,0.60) |
| East Asia | 11218.20(8526.95,14514.12) | 4.86(3.73,6.48) | 39954.86(30356.20,53028.64) | 5.05(3.80,6.62) | 256.16(170.25,360.74) | 0.14(-0.03,0.32) |
| Eastern Europe | 9429.74(8926.14,9817.59) | 12.15(11.45,12.67) | 22102.58(19832.72,24513.40) | 18.39(16.54,20.34) | 134.39(109.09,160.31) | 1.80(1.68,1.92) |
| Eastern Sub-Saharan Africa | 5034.81(3041.62,6386.97) | 18.72(11.43,23.73) | 11477.21(7400.92,14589.70) | 20.00(13.03,25.16) | 127.96(89.70,186.28) | 0.20(0.18,0.23) |
| High-income Asia Pacific | 4841.25(4546.64,5100.33) | 7.10(6.58,7.47) | 18863.83(16673.90,20317.86) | 7.65(6.80,8.23) | 289.65(255.89,317.48) | 0.77(0.46,1.07) |
| High-income North America | 41724.80(39199.56,43301.59) | 29.90(27.80,31.16) | 49580.16(44575.56,52486.98) | 16.43(14.70,17.43) | 18.83(12.16,25.40) | -1.64(-1.89,-1.39) |
| North Africa and Middle East | 5195.73(3873.51,6601.88) | 8.98(6.62,11.58) | 16720.29(11801.68,20203.32) | 10.05(7.09,12.18) | 221.81(164.36,298.64) | 0.50(0.44,0.56) |
| Oceania | 162.26(111.20,223.55) | 19.40(13.29,26.87) | 507.03(320.71,718.56) | 21.76(13.86,30.59) | 212.48(142.88,318.82) | 0.37(0.32,0.42) |
| South Asia | 11085.69(7834.93,13661.44) | 5.16(3.69,6.42) | 34073.22(28061.24,46489.22) | 6.03(4.98,8.26) | 207.36(134.52,305.94) | 0.43(0.32,0.54) |
| Southeast Asia | 7107.14(4947.56,8377.68) | 8.51(6.03,10.11) | 24285.85(15953.39,29602.06) | 10.86(7.10,13.36) | 241.71(177.30,316.76) | 0.96(0.89,1.04) |
| Southern Latin America | 4685.27(4225.81,5208.88) | 26.86(24.27,29.84) | 8445.80(7276.06,9580.81) | 23.26(20.04,26.33) | 80.26(53.72,107.63) | 0.34(0.04,0.65) |
| Southern Sub-Saharan Africa | 2963.78(2165.13,3917.98) | 35.10(26.02,46.01) | 7418.27(5390.58,8681.00) | 44.25(31.48,51.79) | 150.30(93.34,233.55) | 1.01(0.88,1.14) |
| Tropical Latin America | 6896.58(6496.75,7213.96) | 21.59(20.13,22.69) | 21746.84(19995.19,23131.89) | 21.62(19.72,23.06) | 215.33(197.14,233.21) | 0.53(0.27,0.78) |
| Western Europe | 64658.26(61244.41,67382.44) | 29.56(27.81,30.88) | 86450.23(76254.83,93366.54) | 18.36(16.26,19.81) | 33.70(23.30,42.80) | -1.19(-1.41,-0.98) |
| Western Sub-Saharan Africa | 9405.44(5452.35,12333.94) | 30.51(18.00,39.67) | 24689.40(13076.23,32846.57) | 38.56(20.37,50.45) | 162.50(103.62,248.28) | 0.81(0.78,0.85) |

**Notes.** Abbreviations: ASMR, age-standardized mortality; CI, confidence interval; PC, percentage change; EAPC, estimated annual percentage change; UI, uncertainty interval; PCa, prostatic cancer.

**Table S4**. Mortality-to-incidence ratio among PCa patients from 1990 to 2021.

| **Characteristics** | **Incidence crude population’s rate (95% UI)** | | **Mortality crude population’s rate (95% UI)** | | **MIR ratio (%)** | |
| --- | --- | --- | --- | --- | --- | --- |
|  | **1990** | **2021** | **1990** | **2021** | **1990** | **2021** |
| Global | 18.86(17.9,19.54) | 33.45(30.75,35.37) | 7.89(7.23,8.35) | 10.92(9.65,11.71) | 0.42 | 0.33 |
| High SDI | 78.27(74.9,80.42) | 127.28(118.63,133.34) | 24.26(22.91,25) | 28.3(25.44,29.99) | 0.31 | 0.22 |
| High-middle SDI | 15.86(14.95,16.61) | 41.12(36.48,44.7) | 8.21(7.66,8.67) | 13.84(12.23,15.15) | 0.52 | 0.34 |
| Middle SDI | 5.32(4.56,5.88) | 18.5(15.78,20.98) | 3.59(2.99,4.09) | 8.09(6.7,9.37) | 0.67 | 0.44 |
| Low-middle SDI | 3.92(3.06,4.54) | 9.91(8.24,11.51) | 3.29(2.5,3.88) | 5.98(4.86,7.09) | 0.84 | 0.60 |
| Low SDI | 5.08(3.33,6.42) | 6.39(4.12,7.9) | 4.81(3.13,6.07) | 5.26(3.4,6.48) | 0.95 | 0.82 |
| Andean Latin America | 11.83(9.54,14.75) | 33.98(24.74,46.76) | 9.74(7.96,11.96) | 17(12.62,22.57) | 0.82 | 0.50 |
| Australasia | 110.77(100.82,118.11) | 165.56(140.1,194.99) | 36.15(32.93,38.16) | 34.68(29.3,39.79) | 0.33 | 0.21 |
| Caribbean | 40.88(37.75,45.26) | 99.07(84.79,114.11) | 21.69(19.97,24.37) | 39.45(33.95,45.95) | 0.53 | 0.40 |
| Central Asia | 5.48(5.11,5.83) | 9.76(8.75,10.85) | 3.74(3.48,3.97) | 5.06(4.59,5.58) | 0.68 | 0.52 |
| Central Europe | 23.69(22.44,25.06) | 85.26(77.29,93.56) | 15.34(14.56,16.33) | 35.22(32.2,38.03) | 0.65 | 0.41 |
| Central Latin America | 15.87(15.1,16.5) | 58.64(50.48,67.38) | 7.62(7.21,7.93) | 16.25(14.14,18.33) | 0.48 | 0.28 |
| Central Sub-Saharan Africa | 5.52(3.54,7.29) | 6.62(4,9.06) | 5.32(3.45,7.11) | 5.45(3.29,7.48) | 0.96 | 0.82 |
| East Asia | 2.36(1.78,3) | 12.89(9.48,17.22) | 1.79(1.36,2.32) | 5.31(4.03,7.04) | 0.76 | 0.41 |
| Eastern Europe | 19.64(18.57,20.56) | 79.07(70.7,86.77) | 8.91(8.43,9.27) | 22.98(20.62,25.48) | 0.45 | 0.29 |
| Eastern Sub-Saharan Africa | 5.69(3.41,7.26) | 6.88(4.41,8.81) | 5.32(3.21,6.75) | 5.43(3.5,6.91) | 0.93 | 0.79 |
| High-income Asia Pacific | 12.63(11.84,13.35) | 71.88(63.19,79.48) | 5.65(5.3,5.95) | 20.67(18.27,22.27) | 0.45 | 0.29 |
| High-income North America | 144.08(137.79,149.01) | 173.74(163.56,181.32) | 30.33(28.5,31.48) | 27.24(24.49,28.84) | 0.21 | 0.16 |
| North Africa and Middle East | 4.86(3.6,6.01) | 17.3(12.13,21.01) | 2.99(2.23,3.8) | 5.17(3.65,6.25) | 0.62 | 0.30 |
| Oceania | 5.84(4.03,7.99) | 9.31(5.94,12.94) | 4.78(3.28,6.59) | 7.05(4.46,9.99) | 0.82 | 0.76 |
| South Asia | 2.11(1.49,2.58) | 5.12(4.19,6.99) | 1.95(1.38,2.4) | 3.62(2.98,4.94) | 0.92 | 0.71 |
| Southeast Asia | 3.78(2.63,4.41) | 12.35(8.07,15.19) | 3.08(2.14,3.63) | 6.95(4.56,8.47) | 0.81 | 0.56 |
| Southern Latin America | 25.96(23.21,28.95) | 50.87(43.43,58.87) | 19.32(17.42,21.48) | 25.54(22.01,28.98) | 0.74 | 0.50 |
| Southern Sub-Saharan Africa | 13.49(9.72,17.99) | 27.74(20.7,32.83) | 11.66(8.52,15.41) | 18.92(13.75,22.14) | 0.86 | 0.68 |
| Tropical Latin America | 13.76(13,14.42) | 40.87(38.06,43.31) | 9.14(8.61,9.56) | 19.56(17.99,20.81) | 0.66 | 0.48 |
| Western Europe | 77.91(74.22,81.09) | 146.54(132.84,157.54) | 34.54(32.71,35.99) | 40.22(35.48,43.44) | 0.44 | 0.27 |
| Western Sub-Saharan Africa | 10.23(5.88,13.47) | 12.44(6.54,16.76) | 9.8(5.68,12.85) | 10.33(5.47,13.74) | 0.96 | 0.83 |

**Notes.** Abbreviations: MIR, Mortality-to-incidence ratio; UI, uncertainty interval; PCa, prostatic cancer.

**Table S5.** Changes of DALYs number for PCa due to population-level determinants worldwide and in SDI quintiles from 1990 to 2021.

**Notes.** Abbreviations: SDI, Socio-demographic index; DALYs, Disability-Adjusted Life Years; PCa, prostatic cancer.

| **Location** | **Overall difference** | **Changes due to population-level determinants**  **(% contribute to the total changes)** | | |
| --- | --- | --- | --- | --- |
|  |  | Aging | Population | Epidemiological changes |
| Global | 3994842.32 | 77.65 | 58.59 | -36.24 |
| Middle SDI | 1278576.49 | 60.69 | 35.53 | 3.78 |
| High SDI | 790884.1 | 132.34 | 120.59 | -152.93 |
| Low SDI | 348182.56 | 45.81 | 45.31 | 8.89 |
| Low-middle SDI | 742122.27 | 321.24 | 138.72 | -359.96 |
| High-middle SDI | 827928.25 | 70.16 | 58.34 | -28.5 |

**Table S6.** Frontier DALYs and effective difference by 204 countries and territories in 2021.

| **Location** | **ASDR (95% UI)** | **SDI** | **Frontier DALYs** | **Effective difference** |
| --- | --- | --- | --- | --- |
| China | 76.97(56.59,103.60) | 0.72 | 44.04 | 32.93 |
| South Korea | 93.12(64.77,121.35) | 0.57 | 44.09 | 49.03 |
| Taiwan (Province of China) | 192.07(170.35,214.13) | 0.87 | 44.09 | 147.98 |
| Cambodia | 256.04(159.60,347.39) | 0.47 | 47.07 | 208.97 |
| Indonesia | 221.89(135.59,299.79) | 0.66 | 44.09 | 177.80 |
| Lao | 190.49(121.96,270.59) | 0.49 | 46.25 | 144.24 |
| Malaysia | 183.43(121.43,234.58) | 0.74 | 44.07 | 139.37 |
| Maldives | 105.67(61.98,150.73) | 0.65 | 44.06 | 61.61 |
| Myanmar | 188.79(126.32,261.01) | 0.53 | 44.50 | 144.30 |
| Philippines | 293.35(213.25,391.44) | 0.65 | 44.09 | 249.26 |
| Sri Lanka | 108.42(70.65,153.10) | 0.70 | 44.07 | 64.35 |
| Thailand | 187.84(101.68,269.50) | 0.68 | 44.07 | 143.77 |
| Timor-Leste | 161.06(104.03,223.01) | 0.44 | 49.13 | 111.92 |
| Viet Nam | 64.20(32.70,86.23) | 0.63 | 44.13 | 20.07 |
| Fiji | 381.90(125.55,591.00) | 0.68 | 44.07 | 337.83 |
| Kiribati | 233.86(164.82,327.06) | 0.53 | 44.73 | 189.13 |
| Marshall Islands | 495.20(326.59,665.96) | 0.57 | 44.10 | 451.10 |
| Micronesia | 519.67(362.64,685.18) | 0.59 | 44.06 | 475.61 |
| Papua New Guinea | 348.86(193.92,528.63) | 0.42 | 55.86 | 293.00 |
| Samoa | 249.41(139.37,342.56) | 0.59 | 44.08 | 205.33 |
| Solomon Islands | 409.97(269.80,592.16) | 0.43 | 52.36 | 357.61 |
| Tonga | 633.14(484.29,829.01) | 0.63 | 44.09 | 589.05 |
| Vanuatu | 414.80(295.59,547.58) | 0.47 | 46.88 | 367.92 |
| Armenia | 301.79(254.92,346.75) | 0.70 | 44.08 | 257.71 |
| Azerbaijan | 182.46(130.05,245.22) | 0.69 | 44.09 | 138.38 |
| Georgia | 523.21(450.67,608.88) | 0.73 | 44.09 | 479.12 |
| Kazakhstan | 194.30(165.86,224.83) | 0.73 | 44.08 | 150.22 |
| Kyrgyzstan | 146.05(115.19,179.22) | 0.60 | 44.08 | 101.97 |
| Mongolia | 85.97(61.49,114.38) | 0.62 | 44.08 | 41.89 |
| Tajikistan | 78.15(48.91,122.07) | 0.54 | 44.24 | 33.91 |
| Turkmenistan | 102.53(79.13,132.96) | 0.68 | 44.08 | 58.44 |
| Uzbekistan | 87.69(72.22,104.36) | 0.66 | 44.08 | 43.61 |
| Albania | 253.92(172.04,368.42) | 0.71 | 44.08 | 209.85 |
| Bosnia and Herzegovina | 288.65(189.63,394.91) | 0.72 | 44.08 | 244.56 |
| Bulgaria | 409.16(349.37,470.64) | 0.77 | 44.06 | 365.10 |
| Croatia | 422.36(349.52,509.11) | 0.80 | 44.03 | 378.32 |
| Czechia | 352.67(294.31,412.65) | 0.83 | 44.08 | 308.58 |
| Hungary | 342.87(282.83,403.94) | 0.79 | 44.08 | 298.78 |
| North Macedonia | 299.20(200.81,400.52) | 0.75 | 44.06 | 255.14 |
| Montenegro | 523.71(387.72,702.00) | 0.80 | 44.10 | 479.61 |
| Poland | 444.24(395.26,492.58) | 0.81 | 44.10 | 400.14 |
| Romania | 327.37(262.58,399.03) | 0.77 | 44.09 | 283.28 |
| Serbia | 339.00(230.36,450.09) | 0.79 | 44.09 | 294.90 |
| Slovakia | 393.23(273.97,537.25) | 0.81 | 44.10 | 349.14 |
| Slovenia | 438.84(355.41,534.98) | 0.84 | 44.08 | 394.76 |
| Belarus | 433.51(329.57,568.40) | 0.78 | 44.06 | 389.46 |
| Estonia | 652.76(511.91,805.03) | 0.84 | 44.08 | 608.69 |
| Latvia | 623.04(481.35,767.49) | 0.83 | 44.09 | 578.95 |
| Lithuania | 596.24(478.97,728.61) | 0.86 | 44.10 | 552.14 |
| Republic of Moldova | 314.03(263.90,368.33) | 0.73 | 44.09 | 269.94 |
| Russian Federation | 374.01(330.42,416.77) | 0.81 | 44.07 | 329.94 |
| Ukraine | 317.58(208.93,447.24) | 0.76 | 44.08 | 273.49 |
| Brunei Darussalam | 228.10(163.81,292.41) | 0.81 | 44.07 | 184.02 |
| Japan | 131.07(117.63,141.09) | 0.87 | 44.09 | 86.98 |
| Republic of Korea | 124.04(75.33,159.89) | 0.89 | 44.06 | 79.97 |
| Singapore | 128.69(107.86,150.00) | 0.86 | 44.06 | 84.63 |
| Australia | 351.29(297.40,411.89) | 0.84 | 44.08 | 307.21 |
| New Zealand | 371.07(311.65,432.89) | 0.85 | 44.07 | 327.00 |
| Andorra | 368.01(244.70,545.13) | 0.87 | 44.08 | 323.93 |
| Austria | 304.91(256.34,358.18) | 0.85 | 44.08 | 260.83 |
| Belgium | 307.69(258.49,357.35) | 0.85 | 44.07 | 263.61 |
| Cyprus | 382.22(286.88,491.29) | 0.84 | 44.07 | 338.14 |
| Denmark | 476.91(409.02,543.02) | 0.90 | 44.09 | 432.82 |
| Finland | 354.36(300.94,411.49) | 0.86 | 44.08 | 310.28 |
| France | 325.03(273.60,385.12) | 0.84 | 44.09 | 280.94 |
| Germany | 349.38(298.50,400.79) | 0.90 | 44.07 | 305.30 |
| Greece | 301.99(268.10,337.46) | 0.79 | 44.08 | 257.91 |
| Iceland | 390.43(329.01,460.93) | 0.88 | 44.09 | 346.34 |
| Ireland | 291.52(241.56,344.53) | 0.87 | 44.06 | 247.46 |
| Israel | 179.88(151.81,208.86) | 0.81 | 44.11 | 135.77 |
| Italy | 245.93(220.72,270.26) | 0.81 | 44.07 | 201.86 |
| Luxembourg | 281.12(239.23,323.21) | 0.88 | 44.07 | 237.05 |
| Malta | 194.98(158.67,236.55) | 0.80 | 44.06 | 150.92 |
| Netherlands | 360.05(315.59,408.96) | 0.89 | 44.05 | 316.00 |
| Norway | 397.50(353.13,437.75) | 0.92 | 44.12 | 353.37 |
| Portugal | 332.68(279.11,392.25) | 0.74 | 44.07 | 288.61 |
| Spain | 248.10(211.52,289.90) | 0.77 | 44.07 | 204.03 |
| Sweden | 386.51(319.37,455.36) | 0.89 | 44.08 | 342.43 |
| Switzerland | 326.48(271.95,382.26) | 0.93 | 44.07 | 282.41 |
| United Kingdom | 378.95(348.79,400.66) | 0.86 | 44.07 | 334.88 |
| Argentina | 374.33(322.90,430.65) | 0.72 | 44.11 | 330.22 |
| Chile | 392.22(330.19,456.08) | 0.77 | 44.07 | 348.15 |
| Uruguay | 590.15(504.13,687.75) | 0.72 | 44.07 | 546.07 |
| Canada | 265.92(231.74,302.41) | 0.87 | 44.08 | 221.84 |
| United States of America | 334.57(306.05,363.08) | 0.86 | 44.07 | 290.50 |
| Antigua and Barbuda | 1259.86(1116.50,1423.65) | 0.75 | 44.07 | 1215.79 |
| Bahamas | 1186.79(962.96,1454.10) | 0.81 | 44.07 | 1142.72 |
| Barbados | 867.79(668.36,1086.98) | 0.75 | 44.09 | 823.69 |
| Belize | 600.63(514.23,696.84) | 0.61 | 44.12 | 556.51 |
| Cuba | 705.22(598.15,834.42) | 0.67 | 44.07 | 661.15 |
| Dominica | 1067.87(713.93,1495.06) | 0.75 | 44.08 | 1023.78 |
| Dominican Republic | 531.21(338.22,875.08) | 0.62 | 44.08 | 487.13 |
| Grenada | 1542.79(1349.06,1750.86) | 0.67 | 44.06 | 1498.73 |
| Guyana | 938.06(704.53,1205.47) | 0.65 | 44.09 | 893.97 |
| Haiti | 902.52(565.27,1233.44) | 0.45 | 49.06 | 853.47 |
| Jamaica | 1003.43(753.18,1297.19) | 0.68 | 44.06 | 959.37 |
| Saint Lucia | 1201.96(965.31,1442.34) | 0.67 | 44.08 | 1157.88 |
| Saint Vincent and the Grenadines | 1325.19(1160.22,1491.68) | 0.64 | 44.08 | 1281.11 |
| Suriname | 631.21(407.18,911.72) | 0.63 | 44.09 | 587.12 |
| Trinidad and Tobago | 920.61(705.90,1162.05) | 0.77 | 44.05 | 876.56 |
| Bolivia (Plurinational State of) | 504.23(325.38,753.15) | 0.60 | 44.08 | 460.14 |
| Ecuador | 373.55(292.48,470.76) | 0.66 | 44.08 | 329.47 |
| Peru | 344.42(229.75,510.63) | 0.66 | 44.05 | 300.37 |
| Colombia | 337.92(274.15,410.90) | 0.66 | 44.07 | 293.85 |
| Costa Rica | 415.86(360.20,476.10) | 0.70 | 44.08 | 371.78 |
| El Salvador | 399.86(302.17,547.05) | 0.56 | 44.11 | 355.76 |
| Guatemala | 383.25(325.30,454.33) | 0.54 | 44.29 | 338.96 |
| Honduras | 331.19(201.45,562.89) | 0.51 | 45.40 | 285.79 |
| Mexico | 310.54(261.50,367.08) | 0.66 | 44.08 | 266.46 |
| Nicaragua | 277.72(201.00,378.07) | 0.52 | 44.72 | 233.01 |
| Panama | 381.93(294.30,475.20) | 0.71 | 44.07 | 337.86 |
| Venezuela (Bolivarian Republic of) | 595.67(448.77,782.56) | 0.60 | 44.09 | 551.58 |
| Brazil | 374.97(347.39,399.19) | 0.65 | 44.08 | 330.89 |
| Paraguay | 432.39(252.51,649.27) | 0.64 | 44.09 | 388.29 |
| Algeria | 46.25(29.09,63.26) | 0.66 | 44.09 | 2.16 |
| Bahrain | 331.41(238.60,441.83) | 0.75 | 44.08 | 287.33 |
| Egypt | 140.53(94.51,181.33) | 0.61 | 44.11 | 96.42 |
| Iran (Islamic Republic of) | 188.35(125.86,221.59) | 0.70 | 44.09 | 144.27 |
| Iraq | 126.86(86.58,181.51) | 0.66 | 44.06 | 82.81 |
| Jordan | 134.39(85.79,185.14) | 0.73 | 44.08 | 90.31 |
| Kuwait | 173.71(133.16,221.18) | 0.85 | 44.07 | 129.64 |
| Lebanon | 313.05(207.39,416.02) | 0.74 | 44.09 | 268.96 |
| Libya | 243.61(139.44,360.43) | 0.73 | 44.09 | 199.53 |
| Morocco | 106.50(54.34,150.88) | 0.56 | 44.08 | 62.42 |
| Palestine | 318.24(248.00,453.72) | 0.63 | 44.10 | 274.14 |
| Oman | 80.94(54.39,109.56) | 0.77 | 44.09 | 36.85 |
| Qatar | 354.99(229.67,526.75) | 0.85 | 44.07 | 310.93 |
| Saudi Arabia | 87.16(57.10,163.55) | 0.82 | 44.04 | 43.12 |
| Syrian Arab Republic | 168.24(116.55,263.41) | 0.62 | 44.07 | 124.17 |
| Tunisia | 131.53(75.00,190.31) | 0.68 | 44.07 | 87.46 |
| Turkey | 283.29(182.05,382.88) | 0.71 | 44.06 | 239.23 |
| United Arab Emirates | 222.81(160.79,329.20) | 0.85 | 44.06 | 178.75 |
| Yemen | 158.42(97.50,226.93) | 0.45 | 49.02 | 109.39 |
| Afghanistan | 145.64(95.53,200.64) | 0.34 | 76.22 | 69.42 |
| Bangladesh | 94.83(51.69,172.23) | 0.49 | 45.90 | 48.93 |
| Bhutan | 92.34(53.34,169.92) | 0.47 | 47.05 | 45.29 |
| India | 96.37(76.67,130.72) | 0.58 | 44.09 | 52.28 |
| Nepal | 89.06(55.04,157.88) | 0.43 | 51.23 | 37.84 |
| Pakistan | 196.53(134.57,277.26) | 0.50 | 45.66 | 150.88 |
| Angola | 486.19(296.55,656.44) | 0.45 | 49.16 | 437.03 |
| Central African Republic | 451.66(252.09,647.76) | 0.31 | 76.92 | 374.74 |
| Congo | 527.07(300.61,700.51) | 0.58 | 44.10 | 482.98 |
| Democratic Republic of the Congo | 444.24(276.84,642.27) | 0.38 | 76.19 | 368.05 |
| Equatorial Guinea | 534.38(282.75,767.59) | 0.66 | 44.06 | 490.33 |
| Gabon | 587.39(312.59,854.33) | 0.63 | 44.07 | 543.33 |
| Burundi | 405.12(201.27,622.03) | 0.29 | 80.13 | 324.99 |
| Comoros | 476.20(260.58,725.00) | 0.48 | 46.50 | 429.70 |
| Djibouti | 530.08(258.11,835.76) | 0.49 | 46.20 | 483.88 |
| Eritrea | 497.53(235.46,706.70) | 0.40 | 76.19 | 421.35 |
| Ethiopia | 154.62(83.80,229.74) | 0.36 | 76.26 | 78.36 |
| Kenya | 305.34(199.84,401.56) | 0.52 | 44.76 | 260.58 |
| Madagascar | 336.35(171.35,513.02) | 0.40 | 76.23 | 260.12 |
| Malawi | 399.40(284.98,538.30) | 0.38 | 76.22 | 323.19 |
| Mauritius | 303.83(273.87,334.08) | 0.72 | 44.07 | 259.76 |
| Mozambique | 198.72(142.46,283.42) | 0.33 | 76.19 | 122.54 |
| Rwanda | 500.79(268.34,739.36) | 0.44 | 49.57 | 451.23 |
| Seychelles | 867.98(641.10,1088.19) | 0.73 | 44.06 | 823.92 |
| Somalia | 388.51(182.48,631.57) | 0.08 | 388.51 | 0.00 |
| United Republic of Tanzania | 432.13(218.61,637.01) | 0.45 | 49.33 | 382.81 |
| Uganda | 1003.78(744.03,1392.15) | 0.42 | 51.64 | 952.14 |
| Zambia | 833.10(344.82,1321.84) | 0.51 | 45.73 | 787.37 |
| Botswana | 763.86(516.89,1014.56) | 0.64 | 44.07 | 719.79 |
| Lesotho | 841.33(546.11,1242.92) | 0.51 | 45.47 | 795.85 |
| Namibia | 716.15(454.43,923.31) | 0.62 | 44.07 | 672.08 |
| South Africa | 732.97(551.75,869.27) | 0.68 | 44.08 | 688.89 |
| Eswatini | 812.86(479.28,1236.00) | 0.59 | 44.11 | 768.75 |
| Zimbabwe | 1069.82(630.41,1390.23) | 0.47 | 46.80 | 1023.01 |
| Benin | 485.67(264.52,665.39) | 0.37 | 76.16 | 409.50 |
| Burkina Faso | 463.78(251.09,642.49) | 0.29 | 80.17 | 383.61 |
| Cameroon | 561.62(268.60,841.28) | 0.48 | 46.53 | 515.10 |
| Cabo Verde | 898.17(538.70,1452.58) | 0.53 | 44.49 | 853.69 |
| Chad | 446.17(233.77,658.63) | 0.24 | 82.86 | 363.31 |
| Côte d'Ivoire | 772.46(512.77,1049.54) | 0.43 | 53.25 | 719.21 |
| Gambia | 123.19(81.37,173.58) | 0.41 | 62.95 | 60.24 |
| Ghana | 642.74(467.49,875.63) | 0.56 | 44.09 | 598.65 |
| Guinea | 331.05(210.81,459.24) | 0.34 | 76.18 | 254.87 |
| Guinea-Bissau | 567.59(293.76,833.56) | 0.35 | 76.21 | 491.38 |
| Liberia | 440.17(222.12,656.42) | 0.35 | 76.24 | 363.93 |
| Mali | 210.47(146.99,281.42) | 0.27 | 81.80 | 128.67 |
| Mauritania | 517.57(261.13,747.13) | 0.50 | 45.75 | 471.82 |
| Niger | 403.32(202.51,642.19) | 0.17 | 152.08 | 251.24 |
| Nigeria | 802.83(357.38,1202.91) | 0.50 | 45.70 | 757.14 |
| Sao Tome and Principe | 388.07(263.54,527.25) | 0.51 | 45.70 | 342.38 |
| Senegal | 560.48(289.73,785.37) | 0.41 | 64.02 | 496.46 |
| Sierra Leone | 440.97(238.45,652.06) | 0.36 | 76.21 | 364.76 |
| Togo | 564.12(274.51,812.23) | 0.41 | 61.75 | 502.37 |
| American Samoa | 858.56(679.20,1122.42) | 0.72 | 44.08 | 814.48 |
| Bermuda | 781.17(644.74,956.59) | 0.82 | 44.07 | 737.10 |
| Cook Islands | 1028.46(806.16,1313.43) | 0.78 | 44.08 | 984.39 |
| Greenland | 215.48(158.52,284.55) | 0.83 | 44.05 | 171.43 |
| Guam | 239.73(191.00,329.06) | 0.80 | 44.05 | 195.68 |
| Monaco | 418.68(309.42,589.88) | 0.91 | 44.09 | 374.60 |
| Nauru | 541.85(338.74,835.74) | 0.63 | 44.08 | 497.78 |
| Niue | 517.58(343.19,724.68) | 0.73 | 44.08 | 473.50 |
| Northern Mariana Islands | 452.90(340.02,586.78) | 0.77 | 44.08 | 408.82 |
| Palau | 496.40(356.22,731.37) | 0.75 | 44.10 | 452.31 |
| Puerto Rico | 357.59(291.12,431.31) | 0.83 | 44.08 | 313.51 |
| Saint Kitts and Nevis | 1517.58(1257.15,1810.32) | 0.75 | 44.08 | 1473.51 |
| San Marino | 213.71(139.89,332.24) | 0.89 | 44.09 | 169.62 |
| Tokelau | 478.91(344.94,637.94) | 0.69 | 44.09 | 434.82 |
| Tuvalu | 463.65(337.86,603.10) | 0.58 | 44.08 | 419.57 |
| United States Virgin Islands | 640.83(389.86,1074.22) | 0.82 | 44.10 | 596.73 |
| South Sudan | 414.32(204.82,637.80) | 0.28 | 81.27 | 333.05 |
| Sudan | 141.54(94.23,207.09) | 0.54 | 44.26 | 97.27 |

**Notes.** Abbreviations: ASDR, age-standardized disability-adjusted life years; SDI, Socio-demographic index; UI, uncertainty interval; DALYs, disability-adjusted life years.
